# Spatiotemporally programmable cascade hybridization of hairpin DNA in polymeric nanoframework for precise siRNA delivery

**DOI:** 10.1038/s41467-021-21442-7

**Published:** 2021-02-18

**Authors:** Feng Li, Wenting Yu, Jiaojiao Zhang, Yuhang Dong, Xiaohui Ding, Xinhua Ruan, Zi Gu, Dayong Yang

**Affiliations:** 1 Frontiers Science Center for Synthetic Biology, Key Laboratory of Systems Bioengineering (MOE), School of Chemical Engineering and Technology, Tianjin University, Tianjin, P.R. China; 2 Department of Cardiac Surgery, Tianjin Union Medical Centre, Tianjin, P.R. China; 3 School of Chemical Engineering, Australian Centre for NanoMedicine, University of New South Wales, Sydney, NSW Australia

**Keywords:** Molecular self-assembly, DNA nanotechnology, Drug delivery

## Abstract

DNA nanostructures have been demonstrated as promising carriers for gene delivery. In the carrier design, spatiotemporally programmable assembly of DNA under nanoconfinement is important but has proven highly challenging due to the complexity–scalability–error of DNA. Herein, a DNA nanotechnology-based strategy via the cascade hybridization chain reaction (HCR) of DNA hairpins in polymeric nanoframework has been developed to achieve spatiotemporally programmable assembly of DNA under nanoconfinement for precise siRNA delivery. The nanoframework is prepared via precipitation polymerization with Acrydite-DNA as cross-linker. The potential energy stored in the loops of DNA hairpins can overcome the steric effect in the nanoframework, which can help initiate cascade HCR of DNA hairpins and achieve efficient siRNA loading. The designer tethering sequence between DNA and RNA guarantees a triphosadenine triggered siRNA release specifically in cellular cytoplasm. Nanoframework provides stability and ease of functionalization, which helps address the complexity–scalability–error of DNA. It is exemplified that the phenylboronate installation on nanoframework enhanced cellular uptake and smoothed the lysosomal escape. Cellular results show that the siRNA loaded nanoframework down-regulated the levels of relevant mRNA and protein. In vivo experiments show significant therapeutic efficacy of using siPLK1 loaded nanoframework to suppress tumor growth.

## Abstract (web summary)

Controlled delivery of siRNA is important for potential clinical applications. Here, the authors report on the use of cascade hybridization of DNA hairpins with ATP aptamers in DNA crosslinked polymer nanoparticles for triggered release of siRNA and demonstrate anticancer applications.

## Introduction

In living cells, the physicochemical characteristics of confinement essentially play pivotal roles, such as stabilizing the fold conformation of biomolecules, enhancing biochemical reactivity, affecting biochemical equilibrium, and achieving simultaneous occurrence of multi-bioprocesses in confined spaces^1–3^. Inspired by this unique cellular feature, scientists have made efforts to promote the advances in confinement on non-living chemical systems^4^. For example, confinement effects have been utilized to accelerate reactions or stabilize transition states^4–6^. In particular, confinement effects on the assembly of biomolecules have attracted increasing interest in their great potential in biomedicine^5,7–11^. In the case of deoxyribonucleic acid (DNA), its hybridization and assembly under confined space at nanoscale have been demonstrated to be more favorable than in bulk media because of nanoconfinement entropic effects on chemical equilibrium^12,13^.

In recent years, DNA has been used as a versatile building-block to assemble functional nanostructures for a wide range of applications due to the unparalleled sequence programmability, molecular recognition, and biological functions^14–21^. In particular, DNA nanostructures have been proven as promising nanocarriers for delivery of nucleic acid drugs^17,22,23^. The isothermal toehold-mediated DNA hybridization chain reaction (HCR) has been developed as an efficient strategy to prepare DNA nanocarriers^24,25^. In HCR, two metastable state species of DNA hairpins can be triggered by an initiator DNA to yield double helix analogous to alternating copolymers^21,24,26^. Spatiotemporally programming assembly of DNA under nanoconfinement via HCR has been proven promising but challenging for the delivery of nucleic acid drugs due to the steric effect and complexity–scalability–error issues^27,28^.

To address the complexity–scalability–error issues, DNA modules have been introduced into synthetic covalent polymers, which are inspired by biological systems that widely adopt incorporating multiple molecular interactions within a system to achieve hierarchical assembly for specific bioprocess^29,30^. For example, Sleiman and coworkers pioneered combining DNA base-pairing with polymer hydrophobic interactions to achieve anisotropic assembly of DNA cages via hydrophobic interactions^31^. Tanja Weil and coworkers constructed polymers grafted DNA origami and realized transformation of the 3D structural information of DNA origami to polymeric materials^32,33^. Willner and coworkers developed smart hydrogels using DNA-grafted polymers^34,35^. Schulman and coworkers prepared HCR programing high-degree swelling DNA/polymer hybrid hydrogel for the fabrication of soft robots and programmable matters^36,37^. Whereas, DNA assembly under polymeric nanoconfinement has not been explored for the delivery of nucleic acid drugs till now. We envision that achieving programmable assembly of DNA via HCR under polymer-mediated nanoconfinement could be a promising strategy to expand the scalability of DNA for fabricating desirable nanocarriers of nucleic acid drugs.

Herein, the HCR of hairpin DNA in polymeric nanoframework is developed to achieve spatiotemporally programmable assembly of DNA under nanoconfinement for precise siRNA delivery. The polymeric nanoframework with DNA as cross-linker is prepared via precipitation polymerization. By using the potential energy stored in the loops of DNA hairpins to overcome the steric effect under nanoconfinement, the cross-linker DNA in the polymeric nanoframework is designed to initiate the cascade hybridization of two DNA hairpins, thus achieving efficient loading of siRNA in the polymeric nanoframework. Moreover, functional groups such as tumor-targeting phenylboronate are easily decorated on the polymeric nanoframework, which can overcome complexity–scalability–error issues of DNA-only nanosystems, improving the in vivo gene delivery efficiency.

## Results and discussion

### Molecular design

**Fig. 1:**
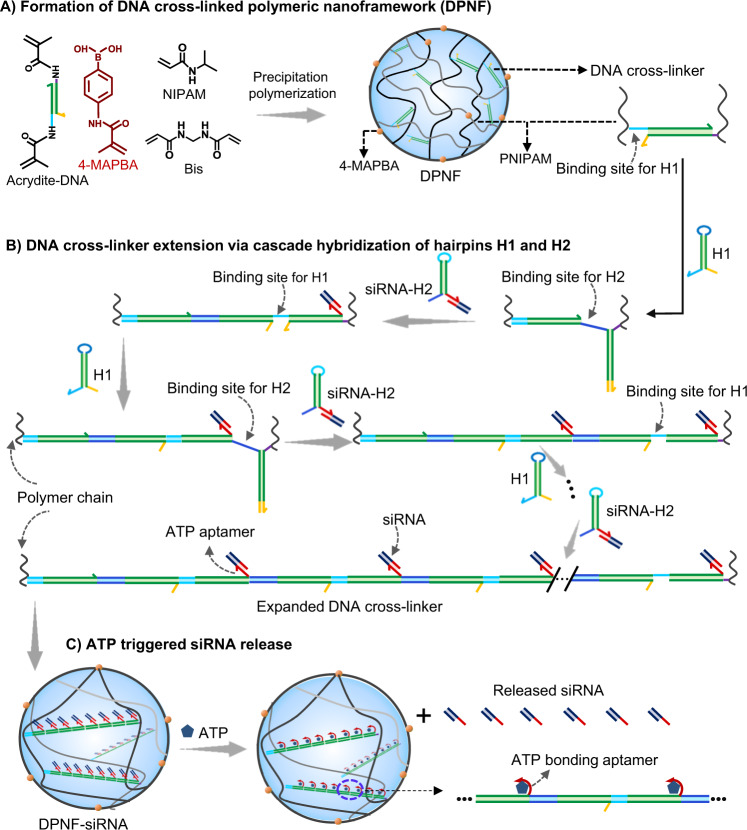
Molecular design. Schematic diagram of **A** the preparation of DPNF, **B** DNA cross-linker extension for siRNA loading via cascade hybridization of DNA hairpins in DPNFs, and **C** ATP-triggered release of siRNA from DPNFs. NIPAM, N-isopropylacrylamide; 4-MAPBA, (4-methacrylamidophenyl) boronic acid; Bis, N,N-methylene diacrylamide; PNIPAM, Poly(N-isopropylacrylamide); ATP, triphosadenine. The single strands with same color in H1 and H2 are complementary sequences.

The DNA cross-linked polymeric nanoframeworks (DPNFs) were prepared via precipitation polymerization of NIPAM (N-isopropylacrylamide), 4-MAPBA ((4-methacrylamidophenyl) boronic acid), Bis (N,N-methylene diacrylamide), and Acrydite-DNA (Fig. 1A). In DPNF, DNA was introduced as cross-linker of the polymer chain and could trigger HCR of H1 and H2 hairpin monomers that were featured with single-stranded toeholds at their 3′ (light blue region) and 5′ ends (dark blue region), respectively (Fig. 1B)^26,36^. The loop region of H1 contained the complemental sequence of H2 toehold, and H2 loop was complementary to H1 toehold, with the double-stranded stems of H1 and H2 being identical (green region). In the absence of trigger DNA, hairpins H1 and H2 could store potential energy in loops and coexist metastably; whereas, upon exposure to the trigger DNA (DNA cross-linker in DPNFs), H1 was opened via toehold-mediated hybridization and strand invasion reaction, making the loop sequence freely accessible. The free loop sequence was then attached to the toehold of H2, thereby initiating a cascade reaction, in which H1 and H2 alternately bonded to each other to form long DNA chain in DPNF (Fig. 1B)^26,36–39^. By elaborately designing an ATP (triphosadenine) aptamer overhang on DNA hairpin H2, siRNA with corresponding complementary overhang could be effectively tethered on DNA hairpin H2 and inserted into the DPNFs with HCR proceeding (Fig. 1B). In response to ATP that was abundant in cells, controlled siRNA release specifically in cellular cytoplasm can be achieved (Fig. 1C)^40–42^. Phenylboronate was introduced onto the DPNFs to enhance cellular uptake via actively recognizing the over-expressed sialic acid residues on tumor cell membrane, and to smooth the lysosomal escape by virtue of acidic pH-responsive transformation from a negatively charged tetravalent hydrophilic form to an uncharged trivalent hydrophobic form^43^. The subtle integration of DNA and synthetic polymer in the DPNF system was anticipated to help achieve in vivo precise siRNA delivery.

### Preparation of DPNFs and HCR of DNA hairpins in DPNF for siRNA loading

**Fig. 2:**
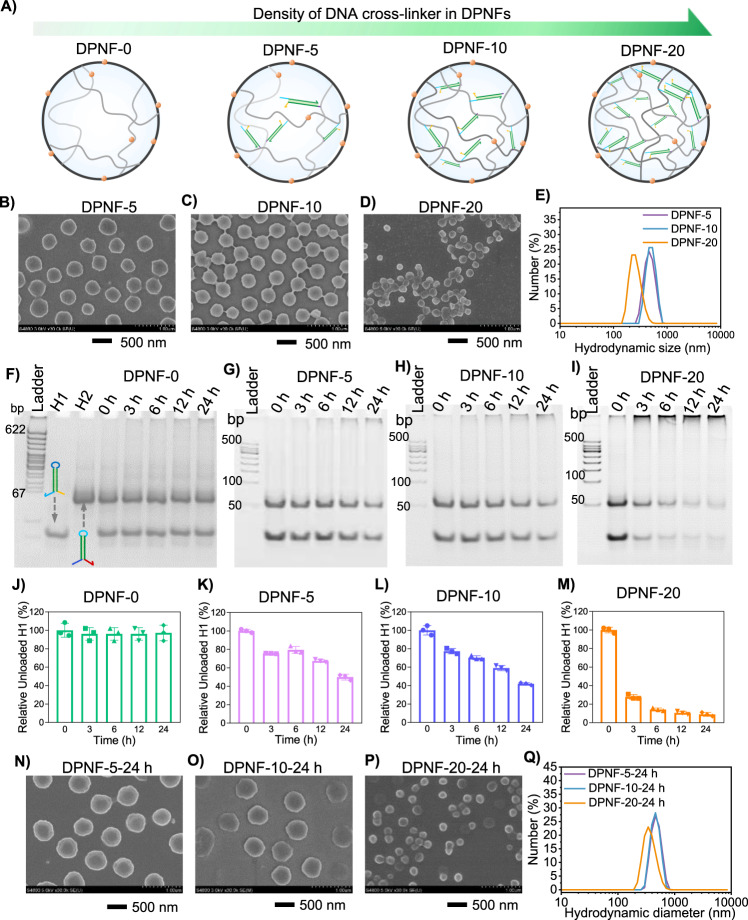
Preparation of DPNF and in situ HCR of H1 and H2 in DPNF. **A** Schematic illustration of DPNFs with varied density of DNA cross-linkers. **B**–**E** SEM images and DLS analysis of DPNF-5, DPNF-10, and DPNF-20. **F**–**I** Gel electrophoresis analysis of HCR of H1 and H2 in DPNF-0, DPNF-5, DPNF-10, and DPNF-20. The concentration of DPNFs was 4.2 mg/ml. The concentration of H1/H2 was 3 μM. **J**–**M** Quantitative analysis of unloaded H1 versus incubation time in gel electrophoresis shown in (**F**–**I**) using image J software. Error bars represent s.d. (*n* = 3 independent replicates). **N**–**Q** SEM images and DLS analysis of DPNF-5, DPNF-10, and DPNF-20 incubated with H1 and H2 for 24 h.

For the preparation of DPNFs, the molar proportion of 4-MAPBA in the monomers was optimized firstly (Supplementary Figs. 1 and 2)^44^. The molar proportion of 4-MAPBA was varied from 1 to 5%. Scanning electron microscope (SEM) images and dynamic light scattering (DLS) analysis showed that the molar proportion of 4-MAPBA 4% led to the generation of nanoparticles with minimal diameter of ~250 nm (Supplementary Fig. 2). Therefore, the molar proportion of 4-MAPBA in the monomers was set as 4% for the preparation of DPNFs. The cross-linker Acrydite-DNA was prepared via self-assembly of two single-stranded DNA C1 and C2 (Supplementary Table 1), which were partially complementary to each other. The concentrations of Acrydite-DNA were set as 5, 10, and 20 μΜ, yielding DPNFs denoted as DPNF-5, DPNF-10, and DPNF-20, respectively (Fig. 2A). The morphology and size of the DPNFs were analyzed by SEM and DLS. SEM images showed that the diameters of DPNF-5 and DPNF-10 were between 400 and 450 nm, while the diameter of DPNF-20 was below 200 nm (Fig. 2B–D). It corresponded to the DLS results in which the average hydrodynamic diameter of DPNF-20 was 266.5 ± 10.2 nm and much smaller than that (~460 nm) of DPNF-5 and DPNF-10 (Fig. 2E). It was speculated that the hydrophilic Acrydite-DNA, as the cross-linker in the DPNF, had significant effect on the polymerization process, cross-linking degree, and nucleation formation of the nanoparticles, and consequently resulted in different sizes of DPNFs.

The two hairpins H1 and H2 used for in situ HCR in DPNFs were designed with 18 intramolecular base pairs in stem and 6 base pairs in loop (Supplementary Table 1). Gel electrophoresis analysis was performed and the results showed that they could coexist at 37 °C (Supplementary Fig. 3). To evaluate the HCR capacity of H1 and H2, theoretical calculation was firstly performed and the results showed that the Gibbs free energies of hybridizations between H1 and H2 were −55.51 and −54.51 kcal/mol, respectively, suggesting that HCR was accessible to H1 and H2. To further explore the Acrydite-DNA initiating HCR between H1 and H2, gel electrophoresis was performed. The gel electrophoresis showed smear bands for a mixture of H1, H2, and Acrydite-DNA, confirming that the hairpins H1 and H2 formed long DNA chains in the presence of Acrydite-DNA (Supplementary Fig. 3). Moreover, it was noted that a higher molar ratio of Acrydite-DNA to H1/H2 resulted in higher hybridization efficiency of H1 and H2 but lower molecular weight of DNA chains, which can be explained by the fact that initiator triggered free radical polymerization of small organic monomers.

Further, we explored whether the DNA hairpins H1 and H2 could be inserted into the DNA cross-linker in DPNFs via HCR. DPNF-0 (set as control), DPNF-5, DPNF-10, and DPNF-20 were incubated with hairpins H1 and H2 (molar ratio of H1 to H2 was 1:1) for indicated time period, followed by gel electrophoresis analysis (Fig. 2F–I). Gel electrophoresis images showed that, with prolonging incubation time, the amount of free H1 and H2 were sequentially decreased while the DNA trapped in the well was increased for DPNF-5, DPNF-10, and especially DPNF-20. In contrast, for the DPNF-0, the free H1 and H2 bands maintained almost identical and little DNA was observed in the well even at incubation time of 24 h. The quantitative analysis of H1 intensity demonstrated that free H1 was decreased by 50.1%, 58.8%, and 91.0% for DPNF-5, DPNF-10, and DPNF-20, respectively (Fig. 2J–M). In particular, free H1 was decreased by ~72.2% within 3 h for DPNF-20. The results confirmed that the DPNFs could efficiently initiate the HCR of H1 and H2 to insert them in DPNFs.

We then examined the evolution of DPNF in morphology and size after HCR using SEM, transmission electron microscopy (TEM), and DLS. SEM images showed that after HCR of H1 and H2 in DPNFs, the DPNF-5, DPNF-10, and DPNF-20 remained well dispersed with a defined spherical structure (Fig. 2N–P and Supplementary Fig. 4). Moreover, the size of all the DPNFs was increased during 24-h incubation: the diameter of DPNF-5 and DPNF-10 was increased from 300–350 to 450–500 nm, and the diameter of the DPNF-20 was significantly increased from ~155 to ~180 nm (Supplementary Figs. 4 and 5). TEM results showed hollow cores in DPNF-5-24, DPNF-20-24, and especially DPNF-10-24, probably due to the inhomogeneous HCR in DPNFs (Supplementary Fig. 6). DLS results also showed that the hydrodynamic diameter of DPNF-5 and DPNF-10 had a little change, while, the DPNF-20 was significantly increased from 266.5 ± 10.2 to 326.1 ± 35.0 nm (Fig. 2Q). Zeta potential analysis was performed to explore the change of the surface potential, and the results showed that DPNF-5 and DPNF-10 exhibited slight decrease; in contrast, for DPNF-20, the zeta potential changed from −7.0 to −13.0 mV (Supplementary Fig. 7).

To explore whether the siRNA could be incorporated into the DPNFs via HCR of DNA hairpins H1 and H2, we firstly designed two single-stranded DNA with one sticky end of 10 bases (denoted as ssDNA-10) or 12 bases (denoted as ssDNA-12) that were complementary to the ATP aptamer overhang of H2 (Supplementary Table 1). The melting temperatures (Tm) for the hybridization of ssDNA-12 and ssDNA-10 with hairpin H2 were theoretically calculated to be 45.9 and 32 °C, respectively. The connection efficiency with H2 was evaluated by gel electrophoresis. The gel electrophoresis results demonstrated that when ssDNA-10 and H2 (molar ratio 1:1) were mixed, two separating bands belonging to ssDNA-10 and H2 were observed, indicating that no hybridization occurred between ssDNA-10 and H2 (Supplementary Fig. 8A). In contrast, when ssDNA-12 and H2 (molar ratio 1:1) were mixed, a new band with lower mobility than ssDNA1 and H2 was observed in the gel electrophoresis, demonstrating the successful hybridization between ssDNA-12 and H2 (Supplementary Fig. 8B). Therefore, the ssDNA-12 was employed as the substitute of siRNA for the following assembly and cell imaging study.

Next, two strategies were employed to assemble ssDNA-12 in DPNFs. In the first strategy (Supplementary Fig. 9A), DPNFs were firstly incubated with H1 and H2 to insert H2 into the DPNFs, and then incubated with ssDNA-12. Gel electrophoresis analysis was performed to evaluate the loading efficiency of ssDNA-12. The results showed that, even after 24-h incubation, the band intensity of ssDNA-12 in DPNF-10 and DPNF-20 group exhibited negligible decrease (Supplementary Fig. 9B-E), demonstrating that little ssDNA-12 was loaded in the DPNF-10 and DPNF-20. It was inferred that the steric effect and electrostatic repulsion between DPNFs and polyanion ssDNA-12 impeded their interaction and further hybridization.

**Fig. 3:**
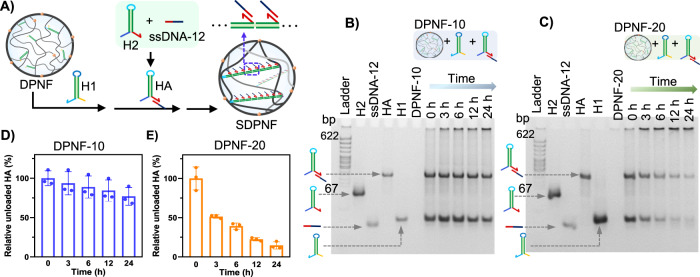
Programmable assembly of ssDNA-12 in DPNF via HCR of DNA hairpins. **A** Schematic description of assembling ssDNA-12 in DPNFs: the ssDNA-12 was linked with H2 to form HA first, and then H1 (3 μM) and HA (3 μM) was co-incubated with DPNFs (0.42 mg/ml). **B** Gel electrophoresis analysis of loading of H1 (3 μM) and HA (3 μM) in DPNF-10 or DPNF-20 (0.42 mg/ml) versus incubation time according to the strategy represented in (**A**). **D**, **E** are quantitative analysis of unloaded HA versus incubation time in (**B**) and (**C**), respectively, using image J software. Error bars represent s.d. (*n* = 3).

Another strategy was the linkage of ssDNA-12 with H2 to form H2-ssDNA-12 (HA) followed by co-incubation of HA and H1 with DPNFs (Fig. 3A). The DPNF-10 and DPNF-20 were, respectively, incubated with H1 and HA. The gel electrophoresis showed that, with prolonging incubation time, the bands belonging to HA and H1 gradually faded and the DNA trapped in the well was increased, demonstrating the successful assembly of HA in the DPNFs (Fig. 3B, C). After 24-h of incubation, the band intensity of HA was decreased by 22.95% and 85.36% for DPNF-10 and DPNF-20, respectively (Fig. 3D, E), confirming that ssDNA-12 was successfully loaded in the DPNFs, and DPNF-20 possessed higher capability for nucleic acid loading than DPNF-10. These results demonstrated that the potential energy stored in the DNA hairpins could overcome the steric effect and electrostatic repulsion to achieve efficient siRNA loading under nanoconfinement.

### Phenylboronic acid (PBA) mediating cellular uptake and lysosomal escape of DPNF

**Fig. 4:**
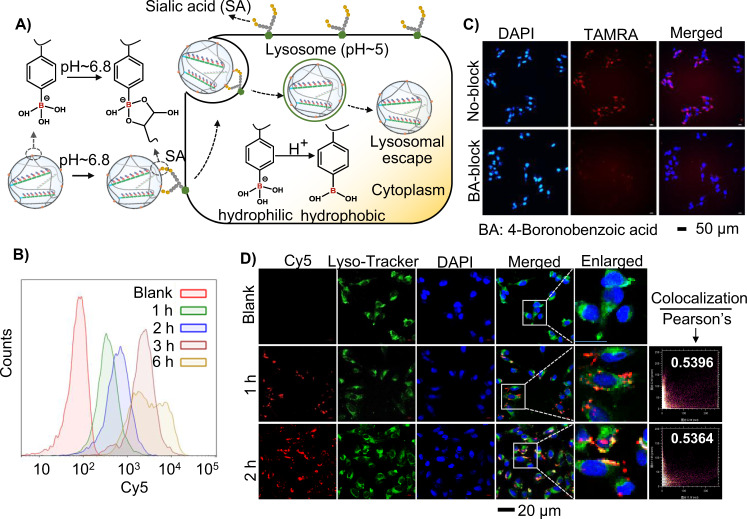
PBA mediating cellular uptake and lysosomal escape of DPNF. **A** Schematic illustration of PBA mediating cellular uptake and lysosomal escape of DPNFs. **B** Flow cytometry analysis of cellular uptake of Cy5-SDPNF-20 (420 μg/ml) by MBA-MD-231 cells at different incubation time. **C** CLSM images of TAMRA-SDPNF-20 treated MBA-MD-231. BA-block group, cells were pretreated with 4-boronobenzoic acid (BA, 0.2 mg/ml) and then incubated with TAMRA-SDPNF-20 (420 μg/ml); No-block group, cells were incubated with TAMRA-DPNF-20 without BA pretreatment. **D** CLSM images of MBA-MD-231 cells incubated with Cy5-DPNF-20 (420 μg/ml) at different time points. Cellular nuclei (blue) were stained by 4′,6-diamidino-2-phenylindole (DAPI) and lysosomes were stained with LysoTracker Green.

Two prerequisites for efficient gene silencing are high nucleic acid drug uptake levels and efficient release in cytoplasm^45,46^. On the membrane of most cancer cells such as MDA-MB-231 (breast cancer cell line) cells, diol containing sialic acid residues is over-expressed. PBA on DPNF-20 could react with sialic acid residues to form annular boronate ester and promote the endocytosis of DPNF-20 (Fig. 4A). The cellular uptake efficiency of DPNF-20 in MDA-MB-231 cells was first evaluated via flow cytometry (FCM) by staining DPNF-20 with Cyanine 5 (Cy5) (Fig. 4B). The FCM analysis results showed significant increase in fluorescence signals in MDA-MB-231 cells with prolonging incubation time, demonstrating continual and effective internalization of Cy5-SDPNF-20. Laser scanning confocal microscope (CLSM) analysis was further performed to explore the mediation function of sialic acid residues in the internalization of DPNF-20. 5-Carboxytetramethylrhodamine (TAMRA) labeled DPNF-20 (denoted as TAMRA-SDPNF-20) was incubated with MDA-MB-231 cells for 6 h, and then CLSM analysis was performed. CLSM images showed significant red fluorescence signals from TAMRA in the cells; whereas, when the cells were pretreated with 4-boronobenzoic acid (BA) that could block the sialic acid receptor before incubation with TAMRA-SDPNF-20, much weaker fluorescence signals from TAMRA were detected in the cells (Fig. 4C). All these results demonstrated an efficient PBA modulating cellular uptake of TAMRA-SDPNF-20^47^.

The unique pH-responsive change of PBA in hydrophobicity facilitated lysosomal escape of PBA decorated nanoparticles. We envisioned that in the acidic lysosome (pH~5), PBA could transform from tetravalent to trivalent, thus resulting in an increase in the hydrophobic PBA fraction and promoting the association of DPNF-20 with lysosomal membranes to realize lysosomal escape^43^. To evaluate the lysosomal escape capability that facilitated by PBA, CLSM analysis of Cy5-DPNF-20 treated MDA-MB-231 cells was performed. The lysosome was stained with LysoTracker Green, and nucleus was stained with DAPI (blue). The overlay of green and red fluorescence demonstrated the Cy5-DPNF-20 that located in lysosomes; the separation of red and green fluorescence demonstrated the Cy5-DPNF-20 that escaped from lysosomes to cytoplasm (Fig. 4D). The Pearson’s correlation coefficients of 1 and 2 h were 0.5396 and 0.5364 quantitatively, demonstrating efficient intracellular lysosomal escape and the dynamic intracellular transport of Cy5-DPNF-20.

### ATP-triggered nucleic acid release from DPNFs

**Fig. 5:**
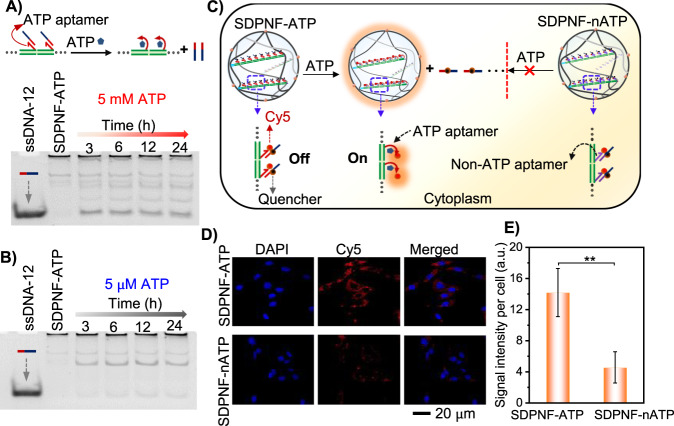
ATP triggered ssDNA-12 release from ATP-responsive DPNF (SDPNF-ATP). **A**, **B** ATP-triggered ssDNA-12 release profiles of SDPNF-ATP in which ssDNA-12 was linked via ATP aptamer sequence. The concentrations of SDPNF-ATP and ssDNA-12 were 4.2 mg/ml and 3 μM, respectively. **C** Schematic illustration of exploration on ATP-triggered ssDNA-12 release from SDPNF-ATP in cytoplasm via FRET. The 3′ overhang of H2 was modified with Cy5 as a fluorescent label and the middle base in ssDNA-12 was modified with BHQ2 as a quencher. SDPNF-nATP, the ssDNA-12 was linked via non-ATP (nATP) aptamer sequence. **D** CLSM images of MDA-MB-231 cells treated with SDPNF-ATP (420 μg/ml) and SDPNF-nATP (420 μg/ml) for 6 h, respectively. **E** Quantitative analysis of fluorescence intensity per cell in SDPNF-ATP group and SDPNF-nATP group. Error bars represent s.d. (*n* = 20 cells), **p < 0.01.

Prior to exploring ATP-triggered nucleic acid release from DPNF, we investigated the ATP-responsive property of HA assembled from ssDNA-12 and H2 with gel electrophoresis. The gel electrophoresis showed that when HA was incubated with 5 mM ATP that corresponded to the intracellular ATP concentrations of 5–10 mM, a band of ssDNA-12 was observed (Supplementary Fig. 10), demonstrating that a portion of ssDNA-12 quickly dissociated from HA. Whereas, still a portion of ssDNA-12 did not dissociate from the HA after 8 h. It was inferred that the competitive binding between ATP and ssDNA-12 with ATP aptamer in H2 was responsible for the incomplete release of ssDNA-12. When ssDNA-12-loaded ATP-responsive DPNF-20 (denoted as SDPNF-ATP) was incubated with 5 mM ATP, a quick release of ssDNA-12 was observed; whereas, no more ssDNA-12 was released even the incubation time was prolonged to 12 h depending on the gray change of ssDNA-12 band (Fig. 5A). The steric effect and electrostatic repulsion between DPNFs and released ssDNA-12 impeded their re-hybridization with DPNFs according to the results in Supplementary Fig. 10. Therefore, it was inferred almost all the ssDNA-12 was released from SDPNF-ATP within 3 h. However, when the concentration of ATP was decreased to 5 μΜ that was higher than the ATP level in plasma (100 nM), only very slight bands belonging to free ssDNA-12 were observed (Fig. 5B), demonstrating that a very small portion of ssDNA-12 was released from SDPNF-ATP. Furthermore, when the ATP aptamer overhang of H2 was replaced with a scramble DNA sequence (non-ATP aptamer, Supplementary Table 1), no ssDNA-12 was released in 5 mM ATP from SDPNF-nATP in which the ssDNA-12 was linked via non-ATP (nATP) aptamer sequence (Supplementary Fig. 11). These results demonstrated the sensitive and specific release capacity of DPNF-20 in response to intracellular ATP.

Furthermore, the ATP-triggered ssDNA-12 release from SDPNF-ATP inside cells was confirmed by using fluorescence resonance energy transfer (FRET). The 3′ overhang of H2 was modified with Cy5 as a fluorescent label and the middle base in ssDNA-12 was modified with black hole quencher BHQ2. In SDPNF, the fluorescence of Cy5 was quenched due to the FRET effect; when ssDNA-12 was released, the fluorescence of Cy5 would be recovered (Fig. 5C). The MDA-MB-231 cells were incubated with labeled SDPNF-ATP and then imaged with CLSM. The labeled SDPNF-nATP was set as control. CLSM images showed significant red fluorescence signals from Cy5 in the SDPNF-ATP treated cells, confirming the effective release of ssDNA-12 in the cells (Fig. 5D). Whereas, the lower level of Cy5 fluorescence signals was detected in the DPNF-nATP treated cells. Quantitative analysis showed ~3.5 times stronger fluorescence signals per cell in the SDPNF-ATP group than that of the SDPNF-nATP group (Fig. 5E). The results demonstrated that the ATP-responsive capacity facilitated the quick release of the target nucleic acid drugs in cytoplasm, which was important for the effective gene silencing effect.

### Gene silencing effect of siRNA-loaded DPNF-20 in vitro

**Fig. 6:**
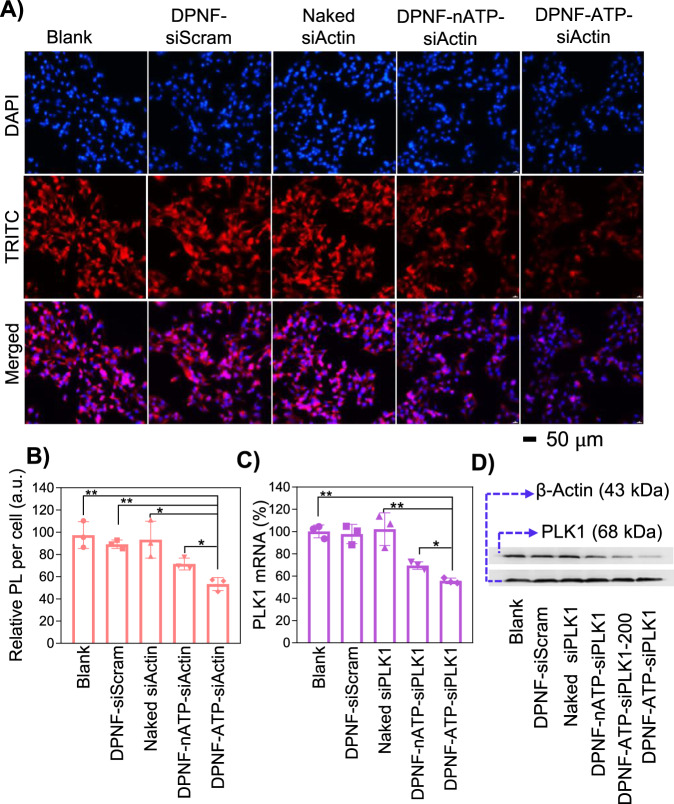
Gene silencing of siRNA-loaded DPNF-20 in vitro. **A** Gene silencing effects of siActin-loaded DPNF-20 in MBA-MD-231 cells. MBA-MD-231 cells were treated, respectively, with naked siActin, DPNF-siScram, DPNF-nATP-siActin, and DPNF-ATP-siActin at same dose for 6 h. After 48 h, the cellular actin cytoskeletons were stained with phalloidin-TRITC and observed with fluorescence microscope. The siActin was 300 nM, and the DPNF was 420 μg/ml. **B** Quantitative analysis of gene silencing effects of siActin in different formulations in (**A**). Error bars represent s.d. (*n* = 3 replicates), *p < 0.05, **p < 0.01. **C**, **D** Gene silencing effect of siPLK1-loaded DPNF-20 in MBA-MD-231 cells. **C** MBA-MD-231 cells were treated, respectively, with naked siPLK1, DPNF-siScram, DPNF-nATP-siPLK1, and DPNF-ATP-siPLK1 with a dose of 300 nM siPLK1 and 420 μg/ml DPNFs. After 48 h, the mRNA levels of PKL1 were measured by RT-qPCR. Error bars represent s.d. (*n* = 3 replicates), *p < 0.05, **p < 0.01. **D** MBA-MD-231 cells were treated with naked siPLK1, DPNF-siScram, DPNF-nATP-siPLK1, and DPNF-ATP-siPLK1, respectively, with a dose of 300 nM, and DPNF-ATP-siPLK1-200 group was treated with a dose of 200 nM siPLK1 and 420 μg/ml DPNFs. After 48 h, the expression of protein PKL1 was analyzed by western blot.

The gene silencing efficiency of siRNA-loaded DPNF-20 was evaluated in MBA-MD-231 cells by using actin gene as the target gene. The silence effect in terms of expression of cellular actin cytoskeletons was confirmed with Lipo3000 as transfection reagent (Supplementary Fig. 12). MBA-MD-231 cells were incubated with different formulations of siActin, i.e., naked siActin, DPNF-siScram (DPNF-20 loaded with scramble RNA sequence), DPNF-nATP-siActin (siActin was linked with non-ATP aptamer in DPNF), and DPNF-ATP-siActin (siActin was linked with ATP aptamer in DPNF) at the same dose (300 nM) of siActin for 6 h. After another 44-h incubation, the cellular actin cytoskeletons were stained with phalloidin-TRITC (red fluorescence) and imaged with fluorescence microscope. Red fluorescence signals of the naked siActin group and the DPNF-siScram group are comparable to the blank group. However, the DPNF-nATP-siActin and DPNF-ATP-siActin treated groups showed weaker signals than the blank group, and the latter group was even weaker than the former one. The results suggested that the DPNF-nATP-siActin and DPNF-ATP-siActin could downregulate the expression of cytoskeletons (Fig. 6A). The cytoskeleton expression per cell was further quantitatively analyzed according to the fluorescence intensities. The results indicated that exposure to naked siActin or DPNF-siScram did not affect the expression of cytoskeletons (Fig. 6B). In contrast, in cells treated, respectively, with DPNF-nATP-siActin and DPNF-ATP-siActin, the cytoskeletons were significantly reduced by 28.58% and 46.68%, respectively.

In vitro therapeutic effect of siRNA-loaded DPNF-20 was then evaluated by using polo-like kinase 1 (PLK1), a key regulator of cellular proliferation that over-expressed in many malignant cells such as MBA-MD-231 cells, as an oncogenic target for RNA interfere therapy. MDA-MB-231 cells were treated with different formulations of anti-PLK1 siRNAs in culture for 6 h. After another 44-h incubation, the PLK1 mRNA and protein expression were measured by real-time quantitative polymerase chain reaction (RT-qPCR) and western blotting, respectively. RT-qPCR results showed that naked siPLK1 treated group and DPNF-siScram treatment did not change the level of PLK1 mRNA compared to the blank group. Notably, DPNF-nATP-siPLK1 group and DPNF-ATP-siPLK1 group showed 30.53% and 44.36% downregulation of PLK1 mRNA, respectively (Fig. 6C). Consistently, the western blot results showed that, compared with blank groups, no significant downregulation of PLK1 protein was detected in the naked siPLK1 group or DPNF-siScram treated groups (Fig. 6D). However, for DPNF-nATP-siPLK1 and DPNF-ATP-siPLK1-200 treated groups, the expression of PLK1 protein was, respectively, reduced by ~48% and ~80% as shown by the band intensity. Furthermore, it is worth to note that even when the concentration of siPLK1 was reduced to 200 nM in the DPNF-ATP-siPLK1-200 treated group, the expression of PLK1 protein was reduced by ~63%, which was much higher than that of DPNF-nATP-siPLK1 treated group in 300 nM, thus confirming that the rapid release of siRNA in the cytoplasm was a vital factor for effective gene silencing.

### Biocompatibility, tumor targeting, and antitumor effect in vivo

In vitro biocompatibility test of DPNF-ssDNA-12 was performed with standard MTT (3-(4,5-dimethylthiazol-2-yl)-2,5-diphenyltetrazolium bromide) assay. MDA-MB-231 cells were incubated with DPNF-20 in varied concentrations for 24 h, and then the cell viability was tested via MTT assay. The MTT results showed that the cells remained satisfactory viability that was higher than 80%, demonstrating the good biocompatibility of DPNF-20 (Supplementary Fig. 13).

To assess the in vivo biosafety of the DPNFs as siRNA delivery vector, we examined the effects of the DPNF-20 on healthy BALB/c mice with normal immunity. Two groups were studied including saline-treated group as control and DPNF-20-treated group (*n* = 3). On days 1, 4, and 7, drug intravenous injections were carried out and body weight was measured; on day 10 the mice were euthanized, and the serum and major organs were collected. The body weight curves showed negligible change in the DPNF-20-treated group compared to the saline (Supplementary Fig. 14A). To study potential changes in organ morphology, postmortem histopathology of the heart, liver, and kidney was analyzed, and no obvious morphological changes were observed (Supplementary Fig. 14B). To further evaluate the potential effects of the DPNFs on the functions of the heart, liver, and kidney, relative serum biochemical index analysis was performed. The collected biochemical indexes showed negligible difference between DPNF-20-treated group and saline-treated group (Supplementary Fig. 14C). It was proposed that the biodegradability property of the cross-linker DNA facilitated the final degradation of DPNFs in vivo. After the enzymatic degradation of cross-linker DNA, the structure of the carrier materials disassembled to release the polymer chains, which could be degraded to oligomers by reactive oxygen species or enzymes in living cells.

Prior to exploring in vivo targeting ability of DPNF-20, the stability of the DPNF-20 was evaluated by incubating DPNF-20 in FBS-containing cell culture medium via polyacrylamide gel electrophoresis^48,49^. It was noteworthy that, almost all DNA was stuck in the well of the gel within 6 h, and there were still more than 60% DNA was stuck in the well according to the gray analysis of the DNA bands, thus indicating that SDPNF-20 could significantly prevent nuclease attack and kept stability in physiological conditions (Supplementary Fig. 15).

**Fig. 7:**
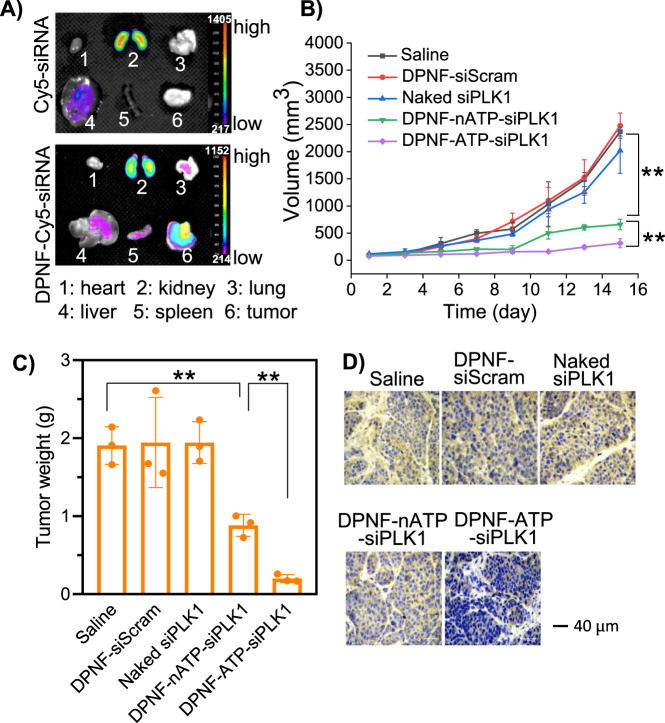
In vivo targeting ability, antitumor, and gene knockdown effects of siPLK1-loaded DPNF in subcutaneous tumor model. **A** In vivo targeting, BALB/c nude mice with subcutaneous xenografts (MDA-MB-231 cells) were injected through tail vein with Cy5-siRNA and DPNF-Cy5-siRNA, respectively. After 24 h, the mice were sacrificed and the tumor, kidney, liver, lung, spleen, and heart were removed for ex vivo imaging. **B** Tumor growth curves. **p < 0.01. **C** Tumor weights measurement. **p < 0.01. **D** Representative immunohistochemical staining of PLK1 protein in tumor tissue in various treatment groups. Data represent mean ± s.d. (*n* = 3 independent mice).

As demonstrated in vitro, the PBA functional group on SDPNF-20 could react with sialic acid residues over-expressed on MDA-MB-231, to form annular boronate ester and promote the active targeting and accumulation of DPNF-20 in the tumor sites. Although PBA can form pH-responsive borate esters with monosaccharides such as glucose in plasma, PBA could form more stable complex with sialic acid residues even in acidic tumor microenvironment that was lower than its pKa, thus leading to selective and high affinity with tumor cells^50,51^. To demonstrate the targeting ability of DPNF-20 in vivo, MBA-MD-231 cells were planted as subcutaneous xenografts in BALB/c nude mice. Mice were injected intravenously with Cy5-labeled siRNA (Cy5-siRNA) and Cy5-siRNA-loaded DPNF-20 (DPNF-Cy5-siRNA), respectively. After 24 h, the mice were sacrificed and the major organs and tumor were collected for ex vivo fluorescence imaging. For the Cy5-siRNA-treated mice, significant fluorescence signals were observed in kidney and liver, but negligible fluorescence signals were observed in the tumor (Fig. 7A). In contrast, for the DPNF-Cy5-siRNA-treated mice, much stronger fluorescence signals at the tumor site was detected compared with kidney and other organs, exhibiting expressive tumor-targeting ability.

To evaluate the antitumor effect, fifteen mice were randomly divided into five groups and intravenously injected with saline, naked siPLK1, DPNF-siScram, DPNF-nATP-siPLK1, and DPNF-ATP-siPLK1 once every 2 days, respectively. The siPLK1 dose was 1 mg/kg per injection. Tumor volumes and body weights of mice were recorded every 2 days, the relative tumor volume was calculated, and the tumor growth and body weight curves with various drug formulation treatments were plotted. Tumor growth curves indicated that the naked siPLK1 and DPNF-siScram groups showed comparable tumor growth with the saline group, while significant tumor suppression was observed in DPNF-nATP-siPLK1 and DPNF-ATP-siPLK1 treatment groups with stronger suppression effect in DPNF-ATP-siPLK1 group (Fig. 7B). Body weight curves showed that the mice in all the treatment groups had no weight change compared to the control group (Supplementary Fig. 16). After 15 days of treatment, the tumor xenografts were excised (Supplementary Fig. 17), and the tumor weights were measured. Compared with saline group, main tumor weight exhibited suppression of 53.68% and 90.47%, respectively, for DPNF-nATP-siPLK1 and DPNF-ATP-siPLK1 treatment groups (Fig. 7C). Furthermore, the cell apoptosis was analyzed by hematoxylin-eosin (H&E) staining and the PLK1 protein expression was analyzed by immunohistochemistry. H&E results showed that both DPNF-nATP-siPLK1 and DPNF-ATP-siPLK1 treatment groups showed patchy necrosis in tumor (Supplementary Fig. 18). Immunohistochemistry analysis showed that, compared with the saline group, the PLK1 protein expression in DPNF-nATP-siPLK1 and DPNF-ATP-siPLK1 treatment groups was downregulated with stronger effect in the DPNF-ATP-siPLK1 treatment group (Fig. 7D). In contrast, the naked siPLK1 and DPNF-siScram treatment did not exhibit significant down-expression of PLK1 in tumor tissues. These results demonstrated that DPNF-ATP-siPLK1 could effectively downregulate the PLK1 expression in vivo given that it could be effectively delivered to tumor sites and released quickly and specifically in cytoplasm.

In conclusion, a DNA HCR-based strategy is developed to achieve spatiotemporally programmable assembly of DNA under nanoconfinement for precise siRNA delivery. In the system, the potential stored in the metastable HCR hairpins succeeded overcoming the steric effect and electrostatic repulsion between the DNA and DPNF to achieve DNA assembly under nanoconfinement and consequently impressive siRNA loading in the DPNF. Moreover, the integration of the unique superiorities of DNA and synthetic polymer overcame the complexity–scalability–error of DNA. By virtue of these features, siRNA loading DPNF with good physiological stability, enhanced cellular uptake, and controlled siRNA release property was obtained; consequently, efficient gene knockdown was achieved both in vitro and in vivo. Given the abundance of DNA-templated reactions and versatile polymer systems, the spatiotemporally programmable assembly of other gene drugs under nanoconfinement is easily achieved, which provides extensible strategy to engineer smart nucleic acid nanoplatform for precision medicine.

## Methods

### Materials

N-isopropylacrylamide (NIPAM) and potassium carbonate (K_2_CO_3_) were purchased from HEOWNS (Tianjin, China). N,N′-Methylenebisacrylamide (Bis), 4-aminophenylboronic acid (4-APBA) and methacryloyl chloride were purchased from Energy Chemical (Shanghai, China). Ammonium persulfate (APS) was purchased from Solarbio Science & Technology Co., Ltd. (Beijing, China). RPMI 1640 medium, fetal bovine serum (FBS), polyvinylidene fluoride (PVDF) membrane with pore size of 0.22 mm, BCA Protein Assay Kit, total RNA Extraction Kit, 3-(4,5-dimethylthiazol-2-yl)-2,5-diphenyltetrazolium bromide (MTT), and 4′,6-diamidino-2-phenylindole (DAPI) were provided by Solarbio Science & Technology Co., Ltd. (Beijing, China). The primary antibodies PLK1 (208G4) Rabbit mAb (#4513T), β-Actin (13E5) Rabbit mAb (#4970T), and horseradish peroxidase-linked secondary antibody Anti-rabbit IgG, HRP-linked antibody (#7074P2) were purchased from Cell Signaling Technology (Shanghai, China).

### DNA and RNA sequences

All DNA oligonucleotides without chemical modifications were synthesized by Shanghai Sangon Biotech Co. Ltd and were purified by polyacrylamide gel electrophoresis. DNA with specially chemical modification was purchased from Shanghai Sangon Biotech Co. Ltd and was purified by HPLC. RNA oligonucleotides were synthesized by Suzhou GenePharma Co. Ltd. All the used oligonucleotide sequences were listed in Supplementary Tables 1 and 2.

### Synthesis of 4-((Acroyloxy)methyl) phenylboronic acid (4-MAPBA)

K_2_CO_3_ (2.70 g, 20 mmol, 8.0 eq.) was added into water (5 mL) under magnetic stirring. 4-Aminophenylboronic acid hydrochloride (0.432 g, 2.5 mmol, 1.0 eq.) was dissolved in acetone (20 mL) and was added into the re-prepared K_2_CO_3_ aqueous solution. Afterward, methacryloyl chloride (0.38 mL, 5 mmol, 2.0 eq.) was added dropwise into the reaction mixture with magnetic stirring at room temperature. After 40 h of reaction, the solvent acetone was removed. The left aqueous solution was acidized utilizing HCl and the product was extracted with ethyl acetate. The ethyl acetate solution was then collected and dried with anhydrous magnesium sulfate. After the ethyl acetate was filtrated and evaporated, precipitation purification was performed to obtain the product. Molecular formula of product: C_9_H_11_BNO_3_, yield: 0.17 g, 0.93 mmol, 38%. The structure of 4-MAPBA was confirmed by the 400 MHz ^1^HNMR (d^6^-DMSO) spectrum (Supplementary Fig. 1).

### Preparation of hairpin structures

Defined stoichiometric amounts of DNA H1 or H2 were added into 1xTAE-Mg^2+^ buffer. The mixtures were heated to 95 °C for 5 min, annealed to 35 °C with a rate of 1 min/°C, and then kept at 35 °C for 2 min to obtain DNA hairpin structures.

### Preparation of Arcydite-DNA

Arcydite-DNA was prepared by mixing equipotent stoichiometric amounts of DNA C1 and C2 in 1xTAE-Mg^2+^ buffer at room temperature.

### Synthesis of DNA cross-linked polymeric nanoframework (DPNF)

NIPAM monomer (65 mM), APS initiator (0.1 wt%), Bis (2 mM), Acry-4-MAPBA (3 mM), and Acrydite-DNA were added into a heart-shaped bottle with continuing N_2_ bubbling. The reaction solution was heated to 70 °C under magnetic stirring for 15 min, and the transparent solution changed to milk-white. The synthesized DPNF was collected by centrifugation (6124 × *g*) and re-suspended in 1xTAE/Mg^2+^ buffer. The final Acrydite-DNA concentrations used were 5, 10, and 20 μM for DPNF-5, DPNF-10, and DPNF-20 preparation, respectively.

### Hybridization chain reaction (HCR) of DNA in DPNF

The DPNFs (4.2 mg/ml) were incubated with H1 and H2 (HA) with a final concentration of 3 µM at 30 °C for different time, and then the nanoparticles were collected by centrifugation (6124 × *g*).

### ATP-triggered release of ssDNA-12 from DPNFs

The ssDNA-12-loaded DPNFs were incubated with ATP with a defined concentration at 37 °C for indicated time. The final concentrations of DPNFs and ssDNA-12 were 4.2 mg/ml and 3 μM, respectively. Gel electrophoresis was performed to analyze the release of target nucleic acid ssDNA-12.

### Agarose gel electrophoresis

Samples were mixed with loading buffer and analyzed by 1% (w/w) agarose with a voltage 6 V/cm in TAE buffer. After electrophoresis, the gel was stained by ethidium bromide (EB, 5 µg/ml), visualized by UV illumination with 312 nm, and photographed by Gel Imaging system.

### Polyacrylamide gel electrophoresis (PAGE)

Twelve percent native PAGE (acrylamide/N,N′-methylenebisacrylamide = 29:1, TBE buffer) was used for sample analysis. After electrophoresis, the gel was stained by EB (5 µg/ml), visualized by UV illumination with 312 nm, and photographed by Gel Imaging system.

### SEM characterization

The cleaned silicon wafers were fixed on a SEM sample stage with conductive adhesive. Afterward, samples were dropped on the fixed silicon wafers and placed in a vacuum oven at 35 °C for 24 h. After drying, the samples were coated with Au, and then were imaged with Hitachi-S4800 FESEM.

### Dynamic light scattering (DLS) measurement

The hydration diameter distribution and zeta potential of the DPNFs were characterized by using a Zetasizer Nano ZS90 (Malvern Instruments, Malvern, UK) with 90° optics and a He-Ne Laser (4.0 mW, 633 nm).

### Stability assay of DPNF

The stability of DPNF-20 was evaluated by incubating DPNF-20 with 10% fetal bovine serum (FBS) containing 1xTAE/Mg^2+^ for different times at 37 °C, which were followed by analysis with 12% PAGE. The DNA hairpins were set as 3 μM, and DPNF-20 was set as 4.2 mg/ml.

### Cell culture

The used human breast cancer cell line MDA-MB-231 was cultured in Dulbecco’s modified Eagle’s medium: 10% fetal bovine serum contains Nutrient Mixture F-12 (DMEM-F-12) at 37 °C in a 5% CO_2_ contained humidified atmosphere.

### Flow cytometry analysis

MDA-MB-231 cells were seeded into 6-well plates and proliferated to around 80–90% confluence. Culture medium was replaced with fresh medium containing Cy5-labeled DPNFs. The final concentration of Cy5 is 3 μM. After incubation for indicated time (1, 2, 4, and 6 h), the cells were washed three times with PBS and harvested by trypsin treatment. Then the harvested cells were washed twice with PBS and collected by centrifugation (861 × *g* for 5 min). Finally, the cells were re-suspended with PBS and lifted with a 300 mesh filter for flow cytometry analysis. A figure exemplifying the gating strategy has been included in the Supplementary information (Supplementary Fig. 19).

### The 4-boronobenzoic acid (BA) blocking assay

MDA-MB-231 cells were seeded onto a 35-mm glass-bottom dishes at a density of 2 × 10^5^ cells/well and cultured at 37 °C for 12 h. The medium was replaced with BA (0.2 mg/ml) contained fresh medium for further incubation of 12 h. Then the medium was replaced with fresh medium containing 5-Carboxytetramethylrhodamine (TAMRA)-labeled DPNF-20 (denoted as TAMRA-SDPNF-20). The concentration of TAMRA-SDPNF-20 was set as 420 μg/ml. Subsequently, the cells were washed with PBS three times and imaged with a fluorescence microscope.

### Lysosomal escape of DPNFs

MDA-MB-231 cells were seeded onto 35 mm glass-bottom dishes at a density of 2 × 10^5^ cells/well and cultured at 37 °C for 12 h. The medium was then replaced with 1 ml of fresh medium containing Cy5-labeled DPNF-20 (Cy5-DPNF-20). The concentration of Cy5-DPNF-20 was set as 420 μg/ml. Intracellular distribution of Cy5-DPNF-20 was analyzed with CLSM. Nuclei and lysosome were, respectively, stained with DAPI and LysoTracker Green dyes.

### Cell viability test

The cell viability was evaluated with standard MTT assay. MDA-MB-231 cells were planted on 96-well plates at a density of 5 × 10^3^ cells per well and incubated for 12 h. Afterward, the cells were incubated with DPNF-20 in varied concentrations at 37 °C for 24 h; then, the cells were washed twice with PBS, and 100 μL of MTT solution (0.5 mg/ml) was added into each well following by a further 4-h incubation. Finally, the primary medium was removed, and 110 μL of DMSO was added. After gently shaking for 10 min, the absorbance at 490 nm (OD 490) of the wells was measured with a microplate reader.

### Real-time quantitative PCR (RT-qPCR) analysis

MDA-MB-231 cells were seeded in six-well plates at a density of 5 × 10^5^ cells per well and incubated for 12 h. Then the cells were treated with naked siPLK1, DPNF-siScram, DPNF-nATP-siPLK1, and DPNF-ATP-siPLK1, respectively. Three hundred nanomolar of siPLK1 was used in the experiments. The concentration of nanoparticles was set as 420 μg/ml. Then the cells were washed by PBS and the intracellular total RNA was extracted with a total RNA Extraction Kit and transcribed reversely into cDNA with a FastKing RT Kit (With gDNase). The quantitative PCR analysis was performed by using SuperReal PreMix Plus (SYBR Green). For β-actin: the forward primer was designed as 5′-ATCGTGCGTGACATTAAGGAGAAG-3′ and the reverse primer was designed as 5′-AGGAAGGAAGGCTGGAAGAGTG-3′; for PLK1, the forward primer was designed as 5′-GGCAACCTTTTCCTGAATGA-3′ and the reverse primer was designed as 5′-AATGGACCACACATCCACCT-3′. The amplification was monitored with LightCycler®480. Data were analyzed with 2^−△△Ct^ method.

### Western blot (WB) assay

MDA-MB-231 cells were seeded in six-well plates at a density of 5 × 10^5^ cells per well and incubated for 12 h. Then the cells were, respectively, cultured with naked siPLK1, DPNF-siScram, DPNF-nATP-siPLK1, and DPNF-ATP-siPLK1 with defined concentration of siPLK1 for 6 h. After 48 h, the proteins were extracted with 1 × SDS Lysis Buffer and quantified with BCA Protein Assay Kit. These proteins were diluted into the same concentration, separated by SDS-PAGE gradient gel, and transferred to the PVDF membrane. Then PVDF membranes were blocked in 5% skimmed milk and incubated with antibodies against PLK1 (1:1000) and β-actin (1:1000). The membrane was incubated with horseradish peroxidase-linked secondary antibody and then analyzed using an automatic chemiluminescence image system (Tanon 4600SF).

### Biosafety assessment

Six mice were equally divided into two groups. One group was treated with DPNF-20 (100 μL, 8 mg·mL^−1^) via intravenously injection. Another group was intravenously injected with saline and was set as control group. The body weights of these mice were recorded for 9 days to explore the physiological influences of DPNF-20 toward organisms. After the treatment, blood of the mice was collected and tests of blood biochemical parameters were performed in Tianjin Medical University General Hospital (China). Besides, the major organs (heart, liver, and kidney) of the mice were extracted, kept in formaldehyde (4%), and stained with hematoxylin and eosin (H&E) for postmortem histopathology study.

### Targeting and distribution in vivo

To study the targeting ability of DPNF in vivo, BALB/c nude mice with breast cancer xenografts were injected with 200 μl of Cy5-labeled naked siRNA (Cy5-siRNA) or Cy5-siRNA-loaded DPNF-20 (DPNF-Cy5-siRNA) through tail vein. The concentrations of siRNA and DPNF-20 were 3 μM and 4.2 mg/ml, respectively. After 24 h, the ex vivo fluorescent images of organs and tumors were obtained by using Berthold Night OWL LB 983 NC100 Imaging system (Berthold, Germany) with an excitation:emission of 650:700 nm.

### Xenograft tumor model

Animal experiments were approved by the ethics committee of Tianjin University in compliance with the law on experimental animals. BALB/c female nude mice (6-week old) were bought from Beijing Huafukang Bioscience Co. Ltd. (Beijing, China). The mice were subcutaneously inoculated with 1 × 10^6^ MDA-MB-231 cells on the right back of the hind leg region and randomly divided into five groups. The control group was intravenously injected with 100 μL saline; naked siPLK1, DPNF-siScram, DPNF-nATP-siPLK1, and DPNF-ATP-siPLK1 at equivalent siPLK1 concentration (a dose of 1 mg/kg) were intravenously injected via the tail vein at initial every 2 days for continuous five times. DPNF-siScram was DPNF-20 loaded with Scramble RNA; DPNF-nATP-siPLK1 was siPLK1-loaded DPNF-20 without ATP-responsive property; DPNF-ATP-siPLK1 was siPLK1-loaded DPNF-20 with ATP-responsive property. Tumor volume was calculated according to the following formula:

1 $$V=L\times W^2 \times 0.5$$

*L* and *W* are the longest and shortest diameters of the tumor, respectively.

### Statistics and reproducibility

All data were reported as mean ± standard deviation (s.d.) from at least three independent runs. The ANOVA *F*-test and Student’s *t* test were used to assess the overall among-group and two-group differences, respectively. In all cases, a *p*-value < 0.05 was considered to be statistically significant. Analyses were performed using Excel2016 analysis software.

### Reporting summary

Further information on research design is available in the Nature Research Reporting Summary linked to this article.

## Supplementary information

Supplementary Information

Reporting Summary

## Data Availability

All data supporting this manuscript are contained within the main text and Supplementary figures. The data collected and reported in this study are available upon request from the corresponding author (including data presented in the main text and in the Supplementary Information).

## References

[1] Mann, S. The origins of life: old problems, new chemistries. *Angew. Chem. Int. Ed.* **52**, 155–162 (2013). 10.1002/anie.201204968 23208616

[2] Dzieciol, A. J. & Mann, S. Designs for life: protocell models in the laboratory. *Chem. Soc. Rev.* **41**, 79–85 (2012). 21952478 10.1039/c1cs15211d

[3] Koga, S., Williams, D. S., Perriman, A. W. & Mann, S. Peptide-nucleotide microdroplets as a step towards a membrane-free protocell model. *Nat. Chem.* **3**, 720–724 (2011). 21860462 10.1038/nchem.1110

[4] Grommet, A. B., Feller, M. & Klajn, R. Chemical reactivity under nanoconfinement. *Nat. Nanotechnol.* **15**, 256–271 (2020). 32303705 10.1038/s41565-020-0652-2

[5] Daube, S. S., Bracha, D., Buxboim, A. & Bar-Ziv, R. H. Compartmentalization by directional gene expression. *Proc. Natl Acad. Sci. USA* **107**, 2836–2841 (2010). 20133663 10.1073/pnas.0908919107 PMC2840370

[6] Rebek, J. Jr. Molecular behavior in small spaces. *Acc. Chem. Res.* **42**, 1660–1668 (2009). 19603810 10.1021/ar9001203

[7] Karzbrun, E., Tayar, A. M., Noireaux, V. & Bar-Ziv, R. H. Programmable on-chip DNA compartments as artificial cells. *Science* **345**, 829–832 (2014). 25124443 10.1126/science.1255550

[8] Lucent, D., Vishal, V. & Pande, V. S. Protein folding under confinement: a role for solvent. *Proc. Natl Acad. Sci. USA* **104**, 10430–10434 (2007). 17563390 10.1073/pnas.0608256104 PMC1965530

[9] Aufinger, L. & Simmel, F. C. Artificial gel-based organelles for spatial organization of cell-free gene expression reactions. *Angew. Chem. Int. Ed.* **57**, 17245–17248 (2018). 10.1002/anie.201809374 PMC6640049 30394633

[10] Guo, X. et al. Gene circuit compartment on nanointerface facilitatating cascade gene expression. *J. Am. Chem. Soc.* **141**, 19171–19177 (2019). 31721571 10.1021/jacs.9b11407

[11] Guo, X. et al. Architectures produced by PCR-based assembly as gene compartments for cell-free gene-expression reactions. *ChemBioChem* **20**, 2597–2603 (2019). 30938476 10.1002/cbic.201900094

[12] Rubinovich, L. & Polak, M. The intrinsic role of nanoconfinernent in chemical equilibrium: evidence from DNA hybridization. *Nano Lett.* **13**, 2247–2251 (2013). 23600497 10.1021/nl4008198

[13] Downs, A. M., McCallum, C. & Pennathur, S. Confinement effects on DNA hybridization in electrokinetic micro- and nanofluidic systems. *Electrophoresis* **40**, 792–798 (2019). 30597594 10.1002/elps.201800356

[14] Yao, C. et al. Double rolling circle amplification generates physically cross-linked DNA network for stem cell fishing. *J. Am. Chem. Soc.* **142**, 3422–3429 (2020). 31893497 10.1021/jacs.9b11001

[15] Wang, D. et al. Transformation of biomass DNA into biodegradable materials from gels to plastics for reducing petrochemical consumption. *J. Am. Chem. Soc.* **142**, 10114–10124 (2020). 32392407 10.1021/jacs.0c02438

[16] Li, F. et al. Preparation of biomimetic gene hydrogel via polymerase chain reaction for cell-free protein expression. *Sci. China Chem.* **63**, 99–106 (2020).

[17] Yuan, Y., Gu, Z., Yao, C., Luo, D. & Yang, D. Nucleic acid-based functional nanomaterials as advanced cancer therapeutics. *Small* **15**, 1900172 (2019). 10.1002/smll.201900172 30972963

[18] Tang, J. et al. Super-soft and super-elastic DNA robot with magnetically driven navigational locomotion for cell delivery in confined space. *Angew. Chem. Int. Ed.* **59**, 2490–2495 (2020). 10.1002/anie.201913549 31769147

[19] Dong, Y. et al. DNA functional materials assembled from branched DNA: design, synthesis, and applications. *Chem. Rev.* **120**, 9420–9481 (2020). 32672036 10.1021/acs.chemrev.0c00294

[20] Li, F., Tang, J., Geng, J., Luo, D. & Yang, D. Polymeric DNA hydrogel: design, synthesis and applications. *Prog. Polym. Sci.* **98**, 101163 (2019).

[21] Song, P. et al. DNA hydrogel with aptamer-toehold-based recognition, cloaking, and decloaking of circulating tumor cells for live cell analysis. *Nano Lett.* **17**, 5193–5198 (2017). 28771008 10.1021/acs.nanolett.7b01006

[22] Li, J. et al. Functional nucleic acid-based hydrogels for bioanalytical and biomedical applications. *Chem. Soc. Rev.* **45**, 1410–1431 (2016). 26758955 10.1039/c5cs00586h PMC4775362

[23] Liu, J., Wang, Z., Zhao, S. & Ding, B. Multifunctional nucleic acid nanostructures for gene therapies. *Nano Res.* **11**, 5017–5027 (2018).

[24] Zhao, Y., Chen, F., Li, Q., Wang, L. & Fan, C. Isothermal amplification of nucleic acids. *Chem. Rev.* **115**, 12491–12545 (2015). 26551336 10.1021/acs.chemrev.5b00428

[25] Liao, W.-C. et al. pH- and ligand-induced release of loads from DNA-acrylamide hydrogel microcapsules. *Chem. Sci.* **8**, 3362–3373 (2017). 28507706 10.1039/c6sc04770j PMC5416914

[26] Fern, J. & Schulman, R. Modular DNA strand-displacement controllers for directing material expansion. *Nat. Commun.* **9**, 3766 (2018). 30217991 10.1038/s41467-018-06218-w PMC6138645

[27] Wijnands, S. P. W., Meijer, E. W. & Merkx, M. DNA-functionalized supramolecular polymers: dynamic multicomponent assemblies with emergent properties. *Bioconj. Chem.* **30**, 1905–1914 (2019). 10.1021/acs.bioconjchem.9b00095 PMC6756584 30860819

[28] Ouyang, C. et al. Precision-guided missile-like DNA nanostructure containing warhead and guidance control for aptamer-based targeted drug delivery into cancer cells in vitro and in vivo. *J. Am. Chem. Soc.* **142**, 1265–1277 (2020). 31895985 10.1021/jacs.9b09782

[29] Cutler, J. I., Auyeung, E. & Mirkin, C. A. Spherical nucleic acids. *J. Am. Chem. Soc.* **134**, 1376–1391 (2012). 22229439 10.1021/ja209351u

[30] Liu, K., Zheng, L., Ma, C., Goestl, R. & Herrmann, A. DNA-surfactant complexes: self-assembly properties and applications. *Chem. Soc. Rev.* **46**, 5147–5172 (2017). 28686247 10.1039/c7cs00165g

[31] Chidchob, P., Edwardson, T. G. W., Serpell, C. J. & Sleiman, H. F. Synergy of two assembly languages in DNA nanostructures: self-assembly of sequence-defined polymers on DNA cages. *J. Am. Chem. Soc.* **138**, 4416–4425 (2016). 26998893 10.1021/jacs.5b12953

[32] Tokura, Y. et al. Bottom-up fabrication of nanopatterned polymers on DNA origami by in situ atom-transfer radical polymerization. *Angew. Chem. Int. Ed.* **55**, 5692–5697 (2016). 10.1002/anie.201511761 27058968

[33] Tokura, Y. et al. Polymer tube nanoreactors via DNA-origami templated synthesis. *Chem. Commun.* **54**, 2808–2811 (2018). 10.1039/c7cc09620h PMC5885267 29492501

[34] Hu, Y. et al. A shape memory acrylamide/DNA hydrogel exhibiting switchable dual pH-responsiveness. *Adv. Funct. Mater.* **25**, 6867–6874 (2015).

[35] Kahn, J. S., Hu, Y. & Willner, I. Stimuli-responsive DNA-based hydrogels: from basic principles to applications. *Acc. Chem. Res.* **50**, 680–690 (2017). 28248486 10.1021/acs.accounts.6b00542

[36] Cangialosi, A. et al. DNA sequence-directed shape change of photopatterned hydrogels via high-degree swelling. *Science* **357**, 1126–1129 (2017). 28912239 10.1126/science.aan3925

[37] Ding, F. et al. A crosslinked nucleic acid nanogel for effective sirna delivery and antitumor therapy. *Angew. Chem. Int. Ed.* **57**, 3064–3068 (2018). 10.1002/anie.201711242 29364558

[38] Zhu, G. et al. Building fluorescent DNA nanodevices on target living cell surface. *Angew. Chem. Int. Ed.* **52**, 5490–5496 (2013). 10.1002/anie.201301439 PMC3755728 23606645

[39] Bi, S., Yue, S. & Zhang, S. Hybridization chain reaction: a versatile molecular tool for biosensing, bioimaging, and biomedicine. *Chem. Soc. Rev.* **46**, 4281–4298 (2017). 28573275 10.1039/c7cs00055c

[40] Mo, R., Jiang, T., DiSanto, R., Tai, W. & Gu, Z. ATP-triggered anticancer drug delivery. *Nat. Commun.* **5**, 3364 (2014). 24618921 10.1038/ncomms4364

[41] Biswas, S. et al. Biomolecular robotics for chemomechanically driven guest delivery fuelled by intracellular ATP. *Nat. Chem.* **5**, 613–620 (2013). 23787753 10.1038/nchem.1681

[42] Xiao, M. et al. Programming drug delivery kinetics for active burst release with DNA toehold switches. *J. Am. Chem. Soc.* **141**, 20354–20364 (2019). 31790242 10.1021/jacs.9b10765

[43] Yoshinaga, N. et al. Polyplex micelles with phenylboronate/gluconamide cross-linking in the core exerting promoted gene transfection through spatiotemporal responsivity to intracellular pH and ATP concentration. *J. Am. Chem. Soc.* **139**, 18567–18575 (2017). 29188718 10.1021/jacs.7b08816

[44] Lamping, S., Otremba, T. & Ravoo, B. J. Carbohydrate-responsive surface adhesion based on the dynamic covalent chemistry of phenylboronic acid- and catechol-containing polymer brushes. *Angew. Chem. Int. Ed.* **57**, 2474–2478 (2018). 10.1002/anie.201711529 29271557

[45] Zhao, X. et al. Co-delivery of HIF1 alpha siRNA and gemcitabine via biocompatible lipid-polymer hybrid nanoparticles for effective treatment of pancreatic cancer. *Biomaterials* **46**, 13–25 (2015). 25678112 10.1016/j.biomaterials.2014.12.028

[46] Whitehead, K. A., Langer, R. & Anderson, D. G. Knocking down barriers: advances in siRNA delivery. *Nat. Rev. Drug Discov.* **8**, 129–138 (2009). 19180106 10.1038/nrd2742 PMC7097568

[47] Zhao, H. et al. Persistent luminescent nanoparticles containing hydrogels for targeted, sustained, and autofluorescence-free tumor metastasis imaging. *Nano Lett.* **20**, 252–260 (2020). 31793303 10.1021/acs.nanolett.9b03755

[48] Wang, D. et al. A molecular recognition approach to synthesize nucleoside analogue based multifunctional nanoparticles for targeted cancer therapy. *J. Am. Chem. Soc.* **139**, 14021–14024 (2017). 28945366 10.1021/jacs.7b08303

[49] Mou, Q. et al. Two-in-one chemogene assembled from drug-integrated antisense oligonucleotides to reverse chemoresistance. *J. Am. Chem. Soc.* **141**, 6955–6966 (2019). 30964284 10.1021/jacs.8b13875

[50] Deshayes, S. et al. Phenylboronic acid-installed polymeric micelles for targeting sialylated epitopes in solid tumors. *J. Am. Chem. Soc.* **135**, 15501–15507 (2013). 24028269 10.1021/ja406406h

[51] Long, Y. et al. Enhanced melanoma-targeted therapy by “Fru-blocked” phenyboronic acid-modified multiphase antimetastatic micellar nanoparticles. *Adv. Sci.* **5**, 1800229 (2018). 10.1002/advs.201800229 PMC6247072 30479911

