# Supplementary material for: Spatiotemporally programmable cascade hybridization of hairpin DNA in polymeric nanoframework for precise siRNA delivery
Source: Nat Commun. 2021 Feb 18;12:1138. doi: 10.1038/s41467-021-21442-7 (PMC7893159; doi:10.1038/s41467-021-21442-7)
Supplement: Supplementary file 1 — Supplementary Information [file 41467_2021_21442_MOESM1_ESM.pdf]

Supplementary information

Spatiotemporally programmable cascade hybridization of hairpin DNA in polymeric nanoframework for precise siRNA delivery

Feng Li[1‡], Wenting Yu[1‡], Jiaojiao Zhang[1], Yuhang Dong[1], Xiaohui Ding[1], Taotao Li[1], Xinhua Ruan[2], Zi Gu[3], Dayong Yang[1]*

[1]Frontiers Science Center for Synthetic Biology, Key Laboratory of Systems Bioengineering (MOE), School of Chemical Engineering and Technology, Tianjin University, Tianjin, 300350, P.R. China.

[2]Department of Cardiac Surgery, Tianjin Union Medical Centre, Tianjin, 300121, P.R. China.

[3]School of Chemical Engineering, Australian Centre for NanoMedicine, University of New South Wales, Sydney, NSW 2052, Australia.

*Corresponding author: dayong.yang@tju.edu.cn

‡These authors contributed equally to this work.

Experimental Sections

Instruments. Scanning electron microscopy (SEM) images were obtained with a Hitachi S-4800 Fesem Field emission scanning electron microscopy (Hitachi, Japan). The htydrodynamic size and zeta potential were performed on a Zetasizer Nano ZS90 unit (Malvern, the United Kingdom). Fluorescent images were obtained using a Biotech Ti-E inverted fluorescence microscope (Nikon, Japan). *In vivo* luminescence images were acquired on a Berthold Night OWL LB 983 NC100 Imaging system (Berthold, Germany). Cell fluorescencent images were collected with LSM 710 confocal microscope (Carl Zeiss, USA); ^{1}H nuclear magnetic resonance (NMR) spectra were collected with liquid NMR spectrometer (AVANCE IIITM HD 400 MHz NanoBAY, Bruker, Germany).

Tables and Figures

Supplementary Table 1. DNA sequences used in this study

Name	Sequences (5'-3')
C1	5`Acrydite-TAAGTTCGCTGTGGCACCTGCACG
C2	5`Acrydite-CAACGTGCAGGTGCCACAGCGTGG
H1	CCACGCTGTGGCACCTGCACGCACCCACGTGCAGGTGCCACAGCG AACTTA
H1-Cy5	5`Cy5- CCACGCTGTGGCACCTGCACGCACCCACGTGCAGGTGCCACAGCG AACTTA
H1-TAMRA	5`TAMRA- CCACGCTGTGGCACCTGCACGCACCCACGTGCAGGTGCCACAGCG AACTTA
H2	TGGGTGCGTGCAGGTGCCACAGCGTAAGTTCGCTGTGGCACCTGC ACGTTGACTCTACCTGGGGGAGTATTGCGGAGGAAGGT
HC	TGGGTGCGTGCAGGTGCCACAGCGTAAGTTCGCTGTGGCACCTGC ACGTTGACTCTACCTGGGGGAGTATTGTCAACCTGCAT
HC-3Cy5	TGGGTGCGTGCAGGTGCCACAGCGTAAGTTCGCTGTGGCACCTGC ACGTTGACTCTACCTGGGGGAGTATTGTCAACCTGCAT-3`Cy5
H2-Cy5	5`Cy5- TGGGTGCGTGCAGGTGCCACAGCGTAAGTTCGCTGTGGCACCTGC ACGTTGACTCTACCTGGGGGAGTATTGCGGAGGAAGGT
H2-3Cy5	TGGGTGCGTGCAGGTGCCACAGCGTAAGTTCGCTGTGGCACCTGC ACGTTGACTCTACCTGGGGGAGTATTGCGGAGGAAGGT-3`Cy5
H2-TAMRA	5`TAMRA- TGGGTGCGTGCAGGTGCCACAGCGTAAGTTCGCTGTGGCACCTGC ACGTTGACTCTACCTGGGGGAGTATTGCGGAGGAAGGT
ssDNA-10-ATP	TGGCAGTGTCTTAGCTGGTTGTACCTTCCTCC
ssDNA−12-ATP	TGGCAGTGTCTTAGCTGGTTGTACCTTCCTCCGC

ssDNA−12-ATP-BHQ2	TGGCAGTGTCTTAGCTGGTTG/iBHQ2dT/ACCTTCCTCCGC
ssDNA-12-nATP	TGGCAGTGTCTTAGCTGGTTGTATGCAGGTTGAC
ssDNA-12-nATP-BHQ2	TGGCAGTGTCTTAGCTGGTTG/iBHQ2dT/ATGCAGGTTGAC
ssDNA-Cy5	TGGCAGTGTCTTAGCTGGTTGTACCTTCCTCCGC-3`Cy5

Red sequences are “sticky ends” as ssDNA linkers. HC was used instead of H2 to connect with nATP DNA/RNA sequences.

Supplementary Table 2. RNA sequences used in this study

Name	Sequences (5’-3’)
siRNA-Actin	Sense:rArCrCrUrUrCrCrUrCrCrGrCrCrGrUrGrCrGrUrGrArCrArUrUrArArGrGrArGrArAdTdT<br>Antisense:rUrUrCrUrCrCrUrUrArArUrGrUrCrArCrGrCrArCrGdTdT
siRNA-nATP-Actin	Sense:rArUrGrCrArGrGrUrUrGrArCrCrGrUrGrCrGrUrGrArCrArUrUrArArGrGrArGrArAdTdT<br>Antisense: rUrUrCrUrCrCrUrUrArArUrGrUrCrArCrGrCrArCrGdTdT
ATP-siPLK1 linker	Sense:rArCrCrUrUrCrCrUrCrCrGrCrUrGrArArGrArArGrArUrCrArCrCrCrUrCrCrUrUrAdTdT<br>Antisense: rUrArArGrGrArGrGrGrUrGrArUrCrUrUrCrUrUrCrAdTdT
nATP-siPLK1 linker	Sense:rArUrGrCrArGrGrUrUrGrArCrUrGrArArGrArArGrArUrCrArCrCrCrUrCrCrUrUrAdTdT<br>Antisense: rArCrGrUrGrArCrArCrGrUrUrCrGrGrArGrArAdTdT
Scramble mimics	Sense:rArCrCrUrUrCrCrUrCrCrGrCrUrUrCrUrCrCrGrArArCrGrUrGrUrCrArCrGrUdTdT<br>Antisense:rArCrGrUrGrArCrArCrGrUrUrCrGrGrArGrArAdTdT

Red sequences are “sticky ends” as siRNA linkers.

Results

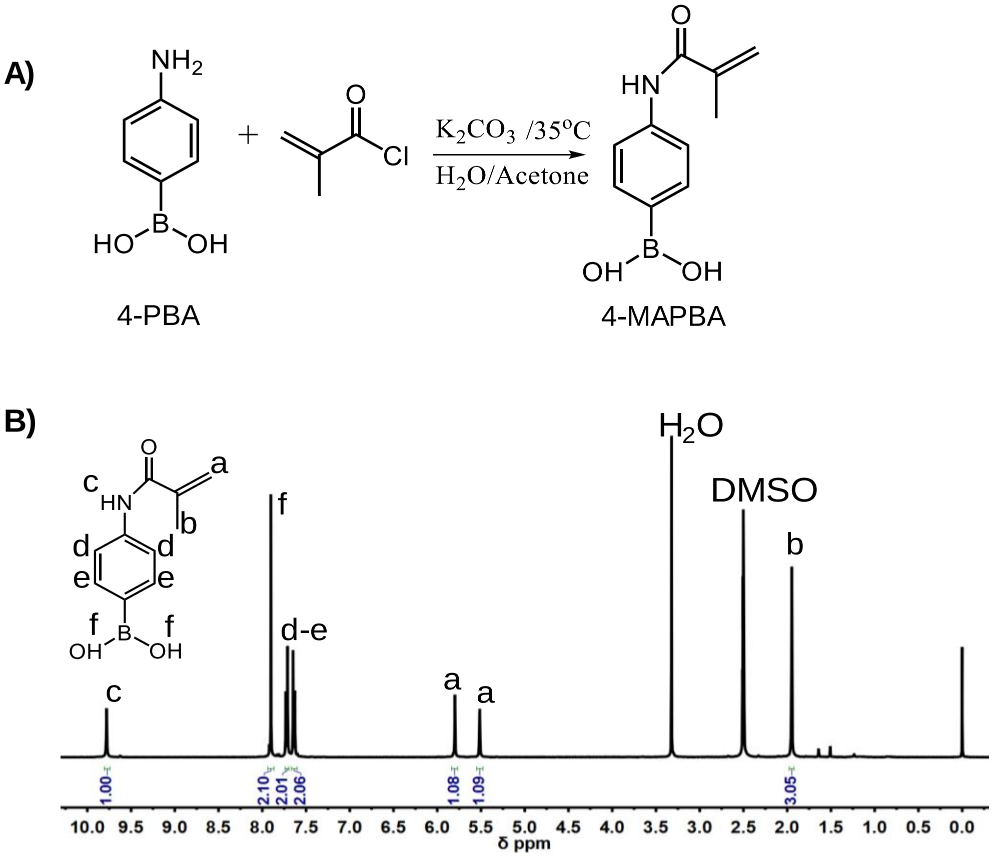

Supplementary Figure 1. A) Synthesis route of (4-methacrylamidophenyl) boronic acid (4-MAPBA).

B) Nuclear ^{1}H magnetic resonance spectrum of the obtained 4-MAPBA.

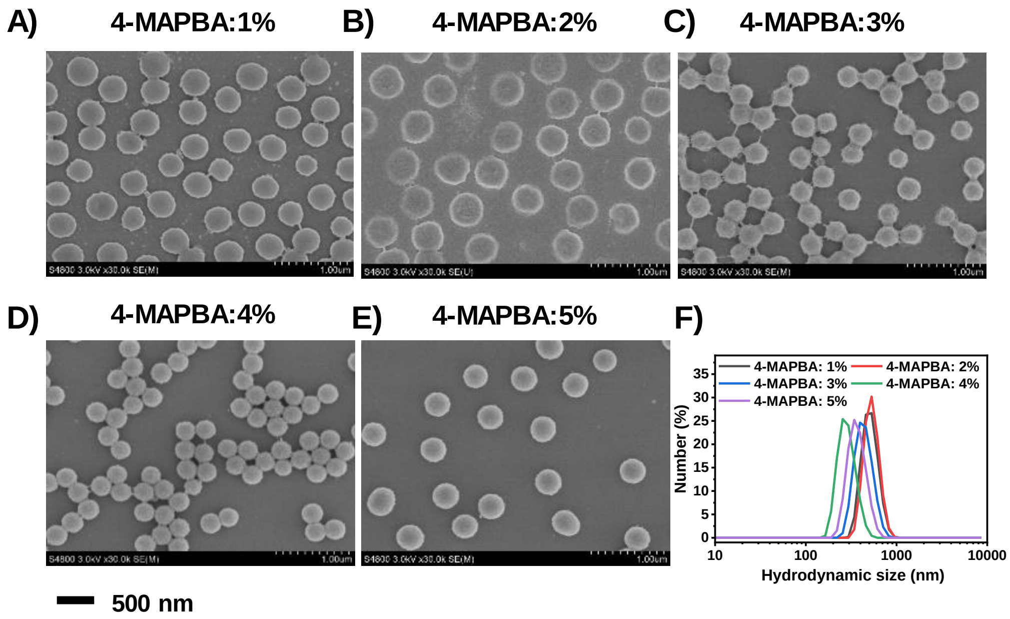

Supplementary Figure 2. A-E) SEM images of Bis cross-linked nanoparticles with varied proportions of 4-MAPBA in the total monomers. **F**) Average hydrodynamic diameter characterized by DLS measurement.

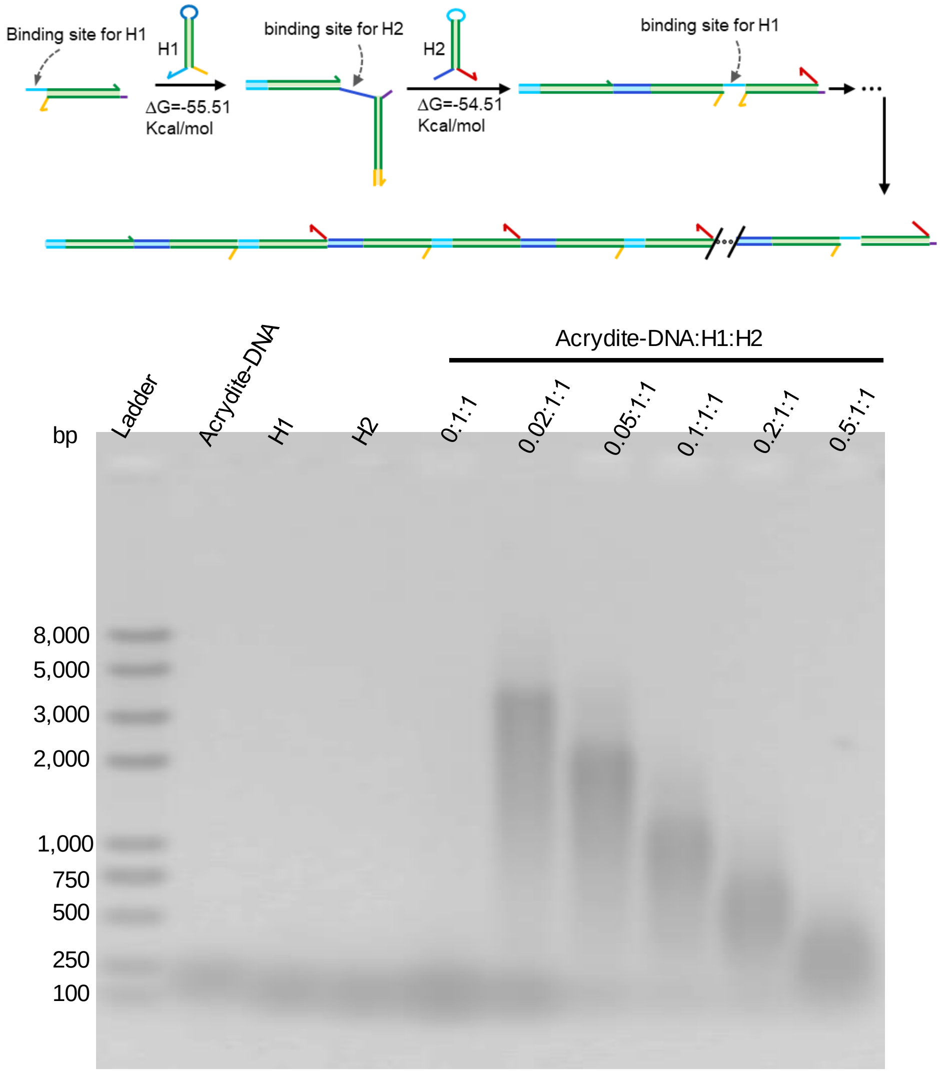

Supplementary Figure 3. Agarose electrophoresis of HCR products of H1 and H2 in the presence of Acrydite-DNA with varied molar ratio of Acrydite-DNA to H1/H2. The molar ratio of H1 to H2 was set as 1.

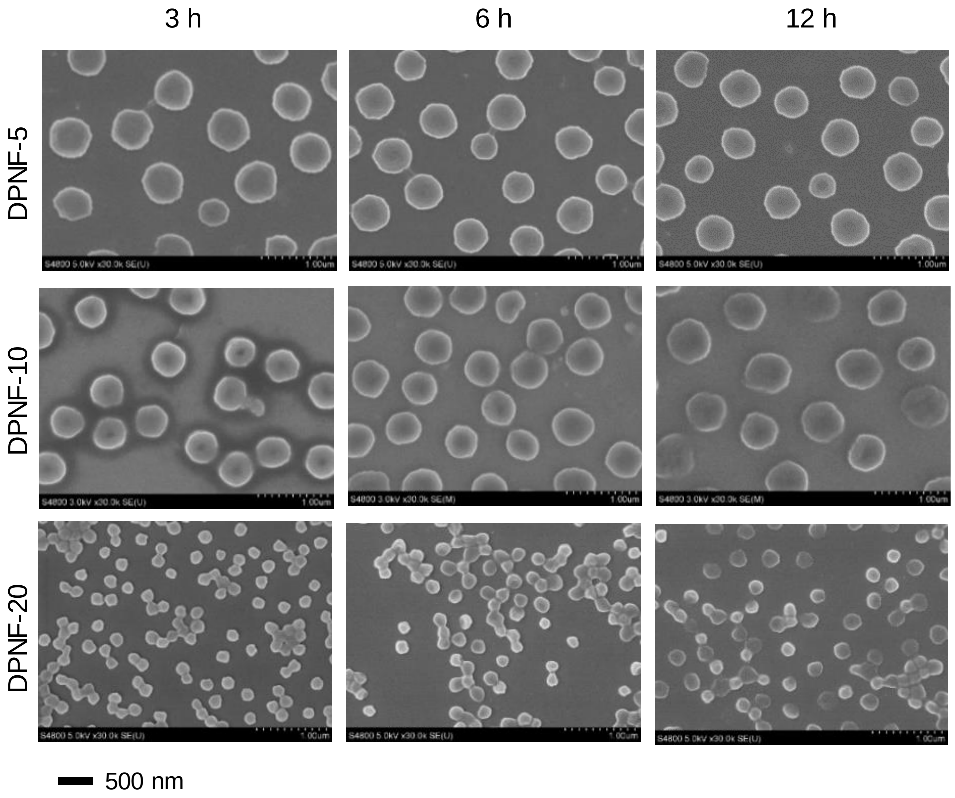

Supplementary Figure 4. Morphology evolution of the DPNFs with prolonging incubation with hairpin DNA H1 and H2.

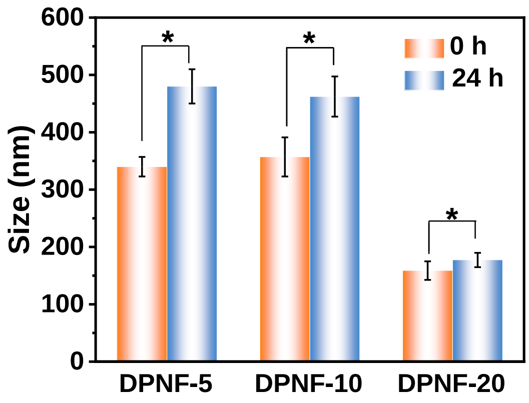

Supplementary Figure 5. Diameter changes of DPNFs after HCR of H1and H2 according to the SEM images. Significant differences were analyzed using image J software. Error bars represent s.d. (n = 20), *P<0.05, (t-test).

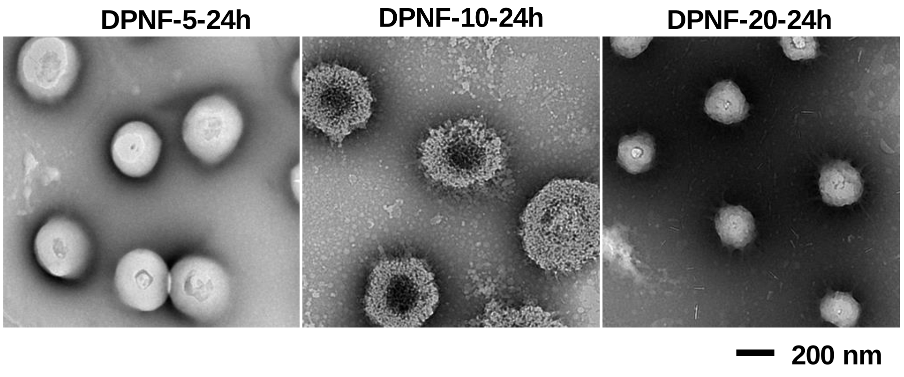

Supplementary Figure 6. Morphology evolution of the DPNFs after incubation with hairpins H1 and H2 for 24 h.

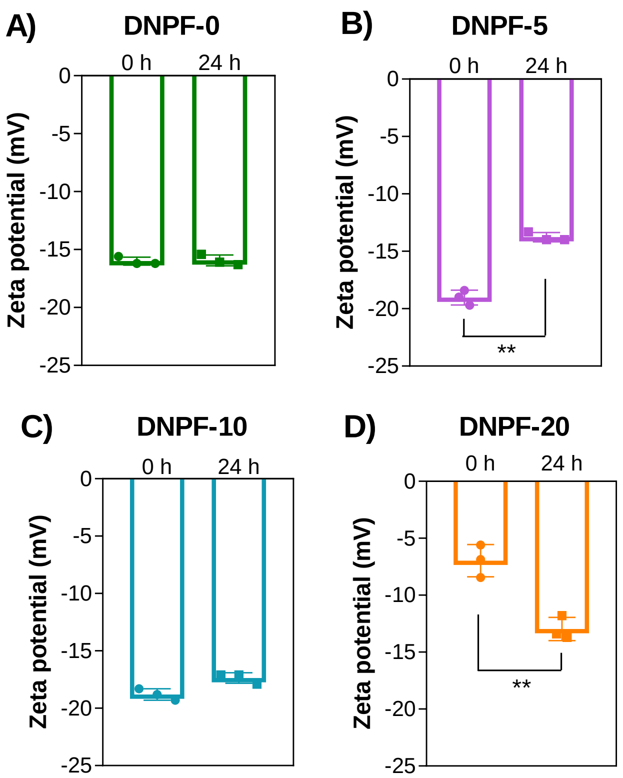

Supplementary Figure 7. Zeta potential of DPNFs before and after incubation with H1and H2 for 24 h. Error bars represent s.d. (n = 3 replicates), ***P*<0.01 (t-test).

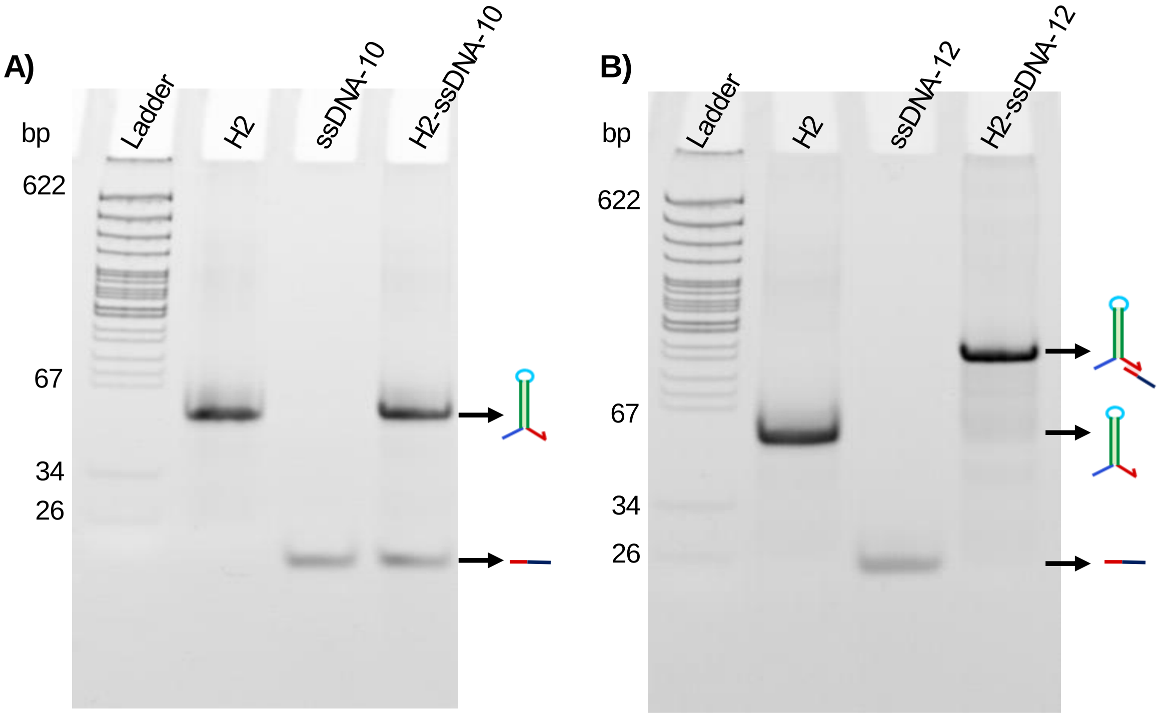

Supplementary Figure 8. Gel electrophoresis analysis of the complementary connection between hairpin H2 and ssDNA-10 and ssDNA-12. **A**) The ssDNA-10 was single-stranded DNA with one sticky end of 10 bases complementary to ATP-specific overhang of H2. **B**) The ssDNA-12 was single-stranded DNA with one sticky end of 12 bases complementary to ATP-specific overhang of H2.

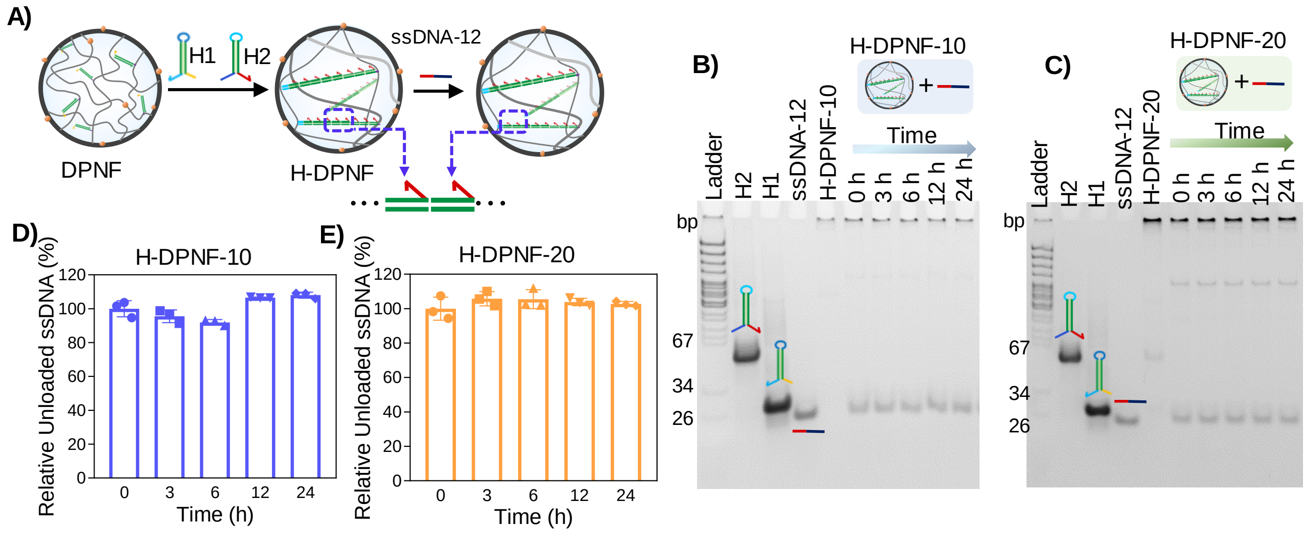

Supplementary Figure 9. A) H1 and HA were co-incubated with DPNFs first and then ssDNA-12 was added to link with HA in the DPNFs. **B**) and **C**) Gel electrophoresis analysis of ssDNA loading in DPNF-10 and DPNF-20 versus incubation time according to the strategy represented in (**A**). **D**) and **E**) Quantitative analysis of unloaded ssDNA-12 versus incubation time in **B**) and **C**) respectively using image J software. Error bars represent s.d. (n = 3).

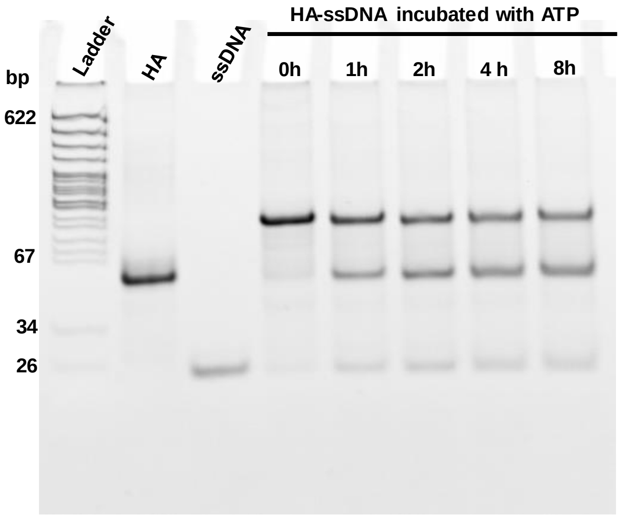

Supplementary Figure 10. Gel electrophoresis image of H2-ssDNA-12 (HA) incubated with ATP (5 mM) at 37°C for indicated time.

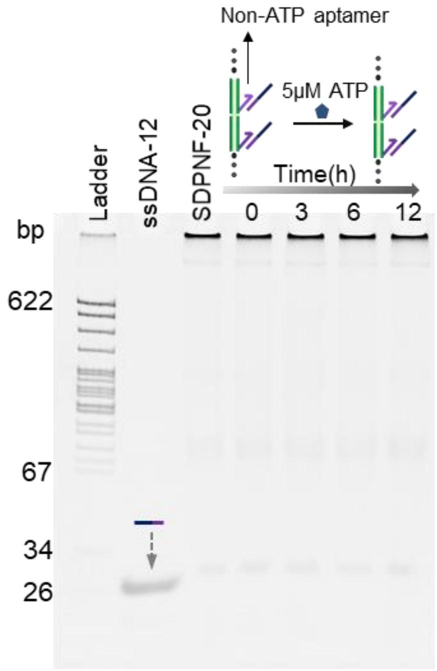

Supplementary Figure 11. Gel electrophoresis image of released ssDNA-12 from DPNF without ATP responsive property (denoted as SDPNF-nATP) in 5 mM ATP solution. In SDPNF-nATP, the ssDNA-12 was linked via non-ATP (nATP) aptamer sequence, and HC was used instead of H2.

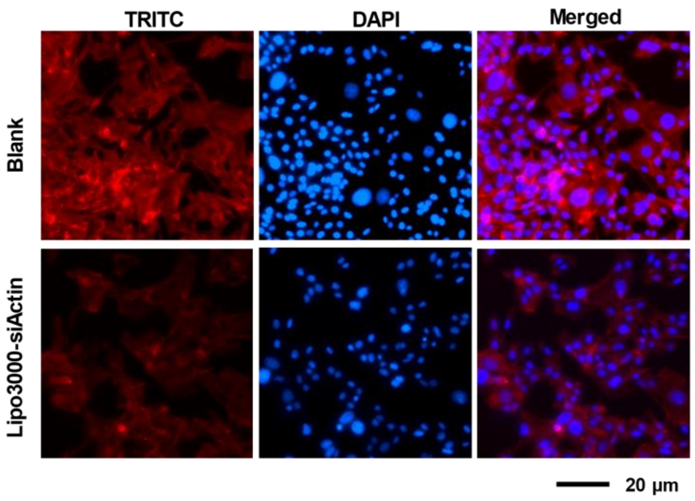

Supplementary Figure 12. Gene silencing of siActin in MDA-MB-231 cells. Lipo3000 was used as transfection reagent, and the concentration of siActin was set as 300 nM. The actin in cellular cytoskeleton was specially stained by phalloidin-TRITC with red fluorescence. The cell nucleus was stained by DAPI (4',6-Diamidino-2-Phenylindole, Dihydrochloride). Compared with Blank group, the Lipo3000-siActin treated group showed much weaker red fluorescence signal, confirming the expression of Actin was effectively silenced.

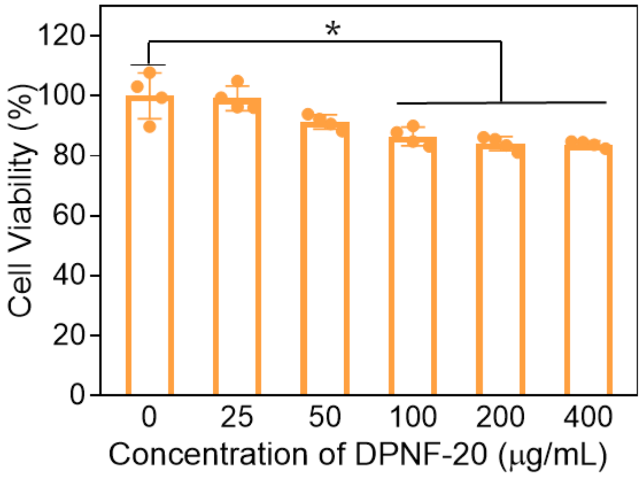

Supplementary Figure 13. Cytotoxicity evaluation of NPNF-20 *in vitro*. MDA-MB-231 cells (breast cancer cell line) were incubated with NPNF-20 in varied concentrations at 37 ^{o}C for 24 h. Afterwards, the viabilities of cells were evaluated by MTT assay. The cellular viability of no treatment was set as 100%. Error bars represent s.d. (n = 4 replicates). *$p < 0.05$ vs (t-test).

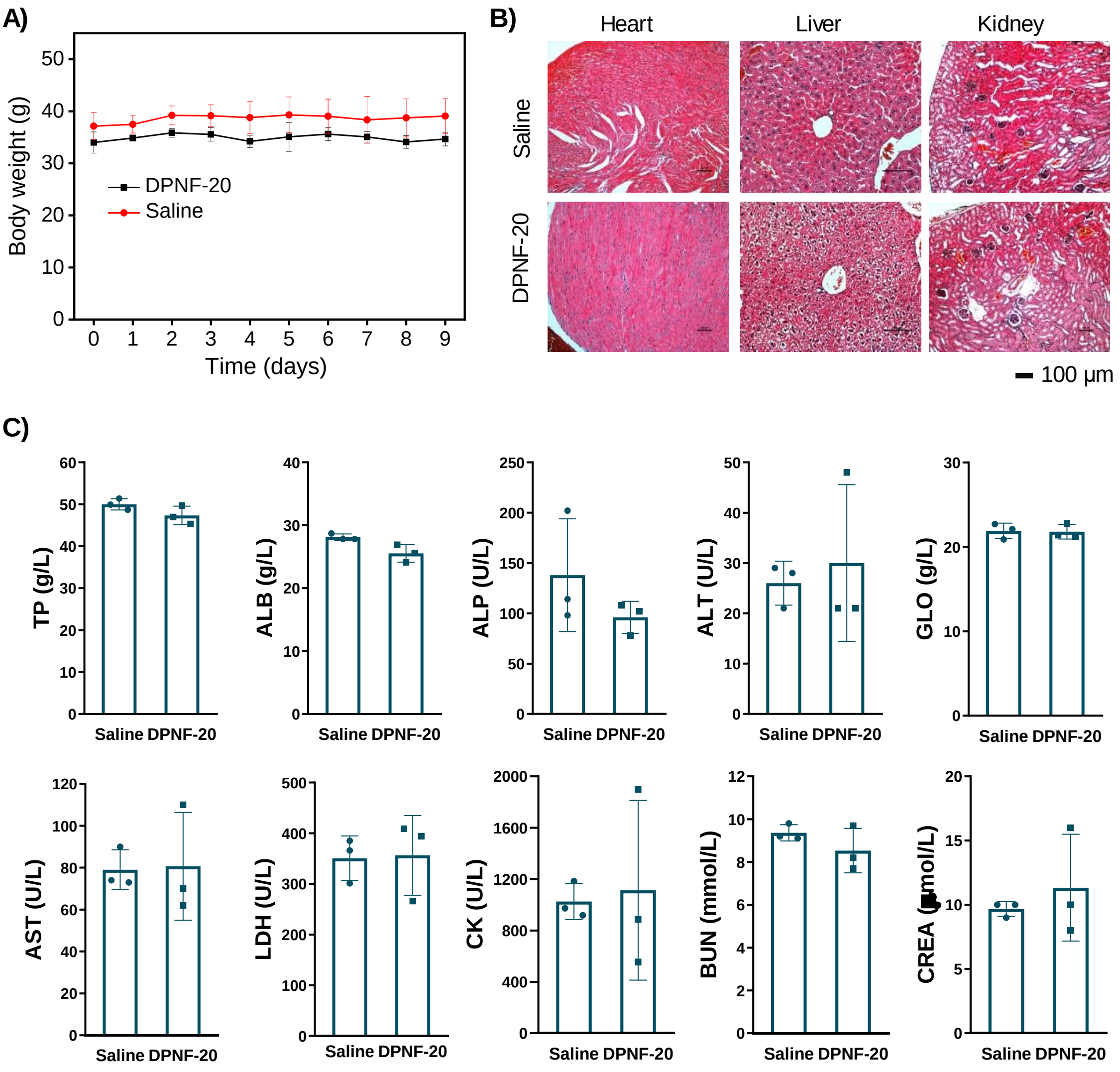

Supplementary Figure 14. Evaluation of potential side effects of the prepared DPNF-20. Healthy BALB/c mice were divided to two groups: saline treated group and DPNF-20 treated group. On day 1, 4, and 7, intravenous injection of drug was carried and body weight was collected; on day 10 the mice were euthanized and the serum and major organs were collected. A) Body weight curves of treated mice as a function of time. Error bars represent s.d. (n = 3). B) Representative H&E staining of heart, liver and kidney. C) Serum biochemical tests (n = 3). TP, total protein; ALB, serum albumin; ALP, alkaline phosphatase; ALT, alanine transaminase; GLO, globulin; AST, aspartate transaminase; LDH, lactate dehydrogenase; CK, creatine kinase; BUN, blood urea nitrogen; CREA, creatinine.

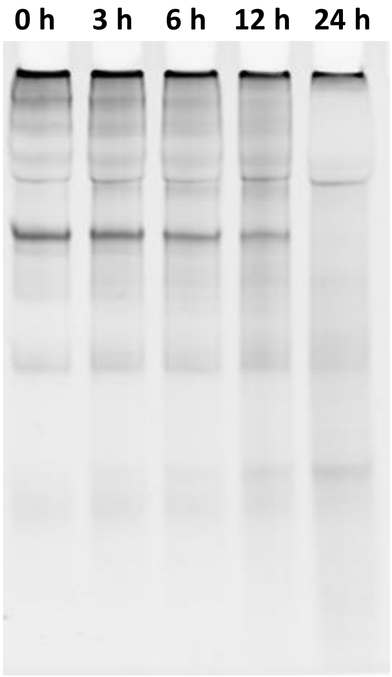

Supplementary Figure 15. Stability assessment of DPNF-20 that was incubated in 10 % fetal bovine serum (FBS) containing medium for different times at 37 °C.

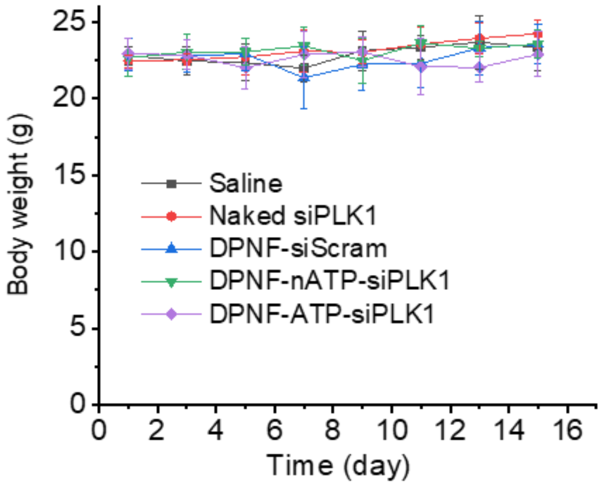

Supplementary Figure 16. Body weight of treated mice as a function of time. DPNF-siScram was DPNF-20 loaded with Scramble RNA; DPNF-nATP-siPLK1 was siPLK1 loaded DPNF-20 without ATP responsive property; DPNF-ATP-siPLK1 was siPLK1 loaded DPNF-20 with ATP responsive property. Data represent mean ± s.d. (n=3).

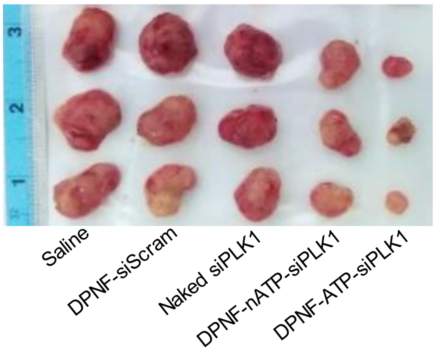

Supplementary Figure 17.Tumors image *ex vivo*. DPNF-siScram was DPNF-20 loaded with Scramble RNA; DPNF-nATP-siPLK1 was siPLK1 loaded DPNF-20 without ATP responsive property; DPNF-ATP-siPLK1 was siPLK1 loaded DPNF-20 with ATP responsive property.

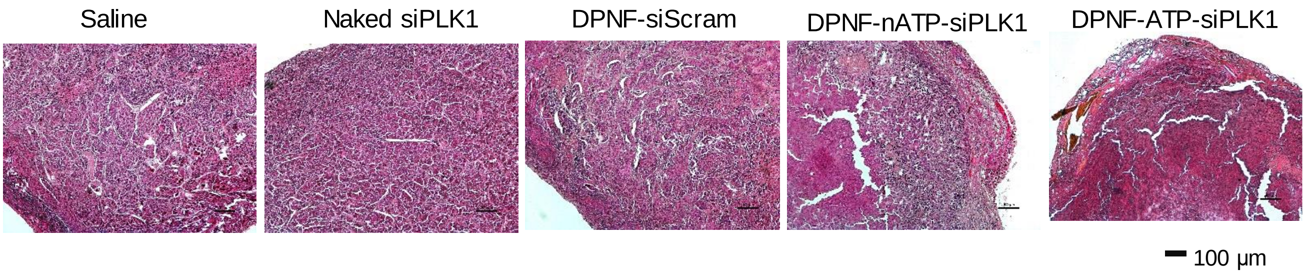

Supplementary Figure 18. Representative H&E staining of tumors in various treatment groups. DPNF-siScram was DPNF-20 loaded with Scramble RNA; DPNF-nATP-siPLK1 was siPLK1 loaded DPNF-20 without ATP responsive property; DPNF-ATP-siPLK1 was siPLK1 loaded DPNF-20 with ATP responsive property.

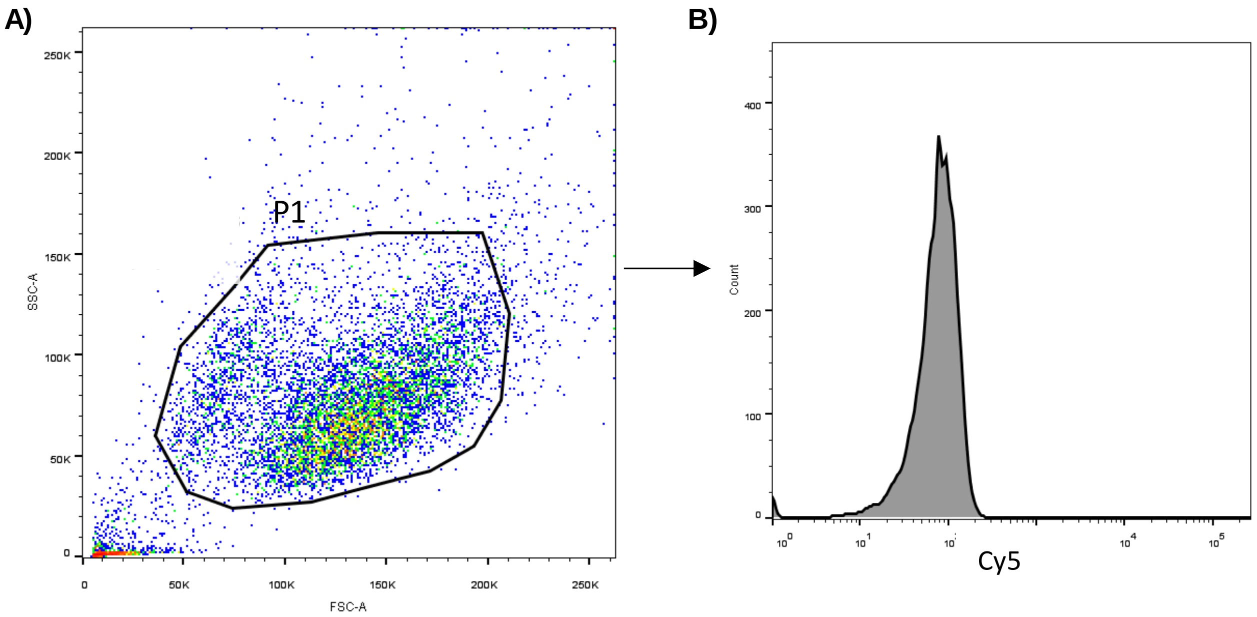

Supplementary Figure 19. The representative gating strategy for flow cytometry analysis. Cells are selected according to the forward scatter (FSC) and side scatter (SSC) area **(A)** parameters. Then the distribution of fluorescence intensity in the cells of gate P1 is determined using APC-Cy5 dye (**B**).
