# Supplementary material for: Spatiotemporally programmable cascade hybridization of hairpin DNA in polymeric nanoframework for precise siRNA delivery
Source: Nat Commun. 2021 Feb 18;12:1138. doi: 10.1038/s41467-021-21442-7 (PMC7893159; doi:10.1038/s41467-021-21442-7)
Supplement: Supplementary file 2 — Reporting Summary [file 41467_2021_21442_MOESM2_ESM.pdf]

nature research

Corresponding author(s): Dayong Yang

Last updated by author(s): Dec 21, 2020

Reporting Summary

Nature Research wishes to improve the reproducibility of the work that we publish. This form provides structure for consistency and transparency in reporting. For further information on Nature Research policies, see our Editorial Policies and the Editorial Policy Checklist.

Statistics

For all statistical analyses, confirm that the following items are present in the figure legend, table legend, main text, or Methods section.

n/a	Confirmed	
☐	☒	The exact sample size (n) for each experimental group/condition, given as a discrete number and unit of measurement
☐	☒	A statement on whether measurements were taken from distinct samples or whether the same sample was measured repeatedly
☐	☒	The statistical test(s) used AND whether they are one- or two-sided<br>*Only common tests should be described solely by name; describe more complex techniques in the Methods section.*
☐	☒	A description of all covariates tested
☐	☒	A description of any assumptions or corrections, such as tests of normality and adjustment for multiple comparisons
☐	☒	A full description of the statistical parameters including central tendency (e.g. means) or other basic estimates (e.g. regression coefficient) AND variation (e.g. standard deviation) or associated estimates of uncertainty (e.g. confidence intervals)
☐	☒	For null hypothesis testing, the test statistic (e.g. F, t, r) with confidence intervals, effect sizes, degrees of freedom and P value noted<br>*Give P values as exact values whenever suitable.*
☒	☐	For Bayesian analysis, information on the choice of priors and Markov chain Monte Carlo settings
☒	☐	For hierarchical and complex designs, identification of the appropriate level for tests and full reporting of outcomes
☐	☒	Estimates of effect sizes (e.g. Cohen's d, Pearson's r), indicating how they were calculated

Our web collection on statistics for biologists contains articles on many of the points above.

Software and code

Policy information about availability of computer code

Data collection	Microsoft PowerPoint 2016, ChemOffice 2018
Data analysis	FlowJo VX10, MestReNova 14, OriginPro 2018, ImageJ Fiji

For manuscripts utilizing custom algorithms or software that are central to the research but not yet described in published literature, software must be made available to editors and reviewers. We strongly encourage code deposition in a community repository (e.g. GitHub). See the Nature Research guidelines for submitting code & software for further information.

Data

Policy information about availability of data

All manuscripts must include a data availability statement. This statement should provide the following information, where applicable:

- Accession codes, unique identifiers, or web links for publicly available datasets
- A list of figures that have associated raw data
- A description of any restrictions on data availability

All data supporting this manuscript are contained within the main text and Supplementary figures. The data collected and reported in this study are available upon request from the correspondence author (including data presented in the main text and in the Supplementary Information).

Field-specific reporting

Life sciences study design

All studies must disclose on these points even when the disclosure is negative.

Sample size	For in vivo experiments, three mice were included in each technical replicate. We did not perform power analysis prior performing these experiments. We chose these sample sizes based on a pilot experiment using three mice in each group to compare the tumor inhibition effect. The experiments demonstrated that three mice were sufficient to detect a significant difference ($p<0.05$) between the nanomedicine treatment group and control group.
Data exclusions	No data was excluded from the analyses.
Replication	All in vitro experiments were replicated independently for at least 3 times. In vivo sample size (n) in each group is detailed in the figure legends or methods section. All attempts at replication were successful.
Randomization	In the reported in vivo experiments, mice were randomly grouped before treatment. As the work does not involve participant groups, randomization was not used for this study.
Blinding	The person who executed the treatment experiments was blinded to another person who carried out measurement of the tumor sizes.

Reporting for specific materials, systems and methods

We require information from authors about some types of materials, experimental systems and methods used in many studies. Here, indicate whether each material, system or method listed is relevant to your study. If you are not sure if a list item applies to your research, read the appropriate section before selecting a response.

Materials & experimental systems

n/a	Involved in the study	
☐	☒	Antibodies
☐	☒	Eukaryotic cell lines
☒	☐	Palaeontology and archaeology
☐	☒	Animals and other organisms
☒	☐	Human research participants
☒	☐	Clinical data
☒	☐	Dual use research of concern

Methods

n/a	Involved in the study	
☒	☐	ChIP-seq
☐	☒	Flow cytometry
☒	☐	MRI-based neuroimaging

Antibodies

Antibodies used	All antibodies used in the figures are listed in the Methods with supplier name and catalog number.<br>Primary antibodies: PLK1 (208G4) Rabbit mAb (#4513T), dilution 1:1000; β-Actin (13E5) Rabbit mAb (#4970T), dilution 1:1000.<br>Horseradish peroxidase-linked secondary antibodies: HRP-linked antibody (#7074P2), dilution, 1:2000.
Validation	According to the manufacturer, there have been more than 5000 citations reporting the reactivity to HRP-linked Antibody (#7074P2), more than 50 citations for PLK1 Antibody (#4513T), and more than 2000 for β-Actin (13E5) Rabbit mAb (#4970T).

Eukaryotic cell lines

Policy information about cell lines

Cell line source(s)	The human breast cancer cell line MDA-MB-231 used in this work was purchased from the Shanghai Institute of Biochemistry and Cell Biology, CAS, China.
Authentication	Authentication of the cell lines by the vendors was confirmed vis STR prior to their purchase and use.
Mycoplasma contamination	The cell lines were tested with Universal Mycoplasma Detection Kit (ATCC #30-1012K) and confirmed free of mycoplasma contamination.
Commonly misidentified lines<br>(See ICLAC register)	No commonly misidentified cell lines were used in the study.

Animals and other organisms

Policy information about studies involving animals; ARRIVE guidelines recommended for reporting animal research

Laboratory animals	Athymic female NU/NU nude mice (6 weeks old) were purchased from Beijing Huafukang Bioscience Co. Ltd. (Beijing, China) for in vivo studies. Animals were housed in an animal facility, where temperature is 23-27◦C , humidity is 40-70%, and a 12 hour light/12

	hour dark cycle is set.
Wild animals	The study did not involve wild animal.
Field-collected samples	The study did not involve samples collected from field.
Ethics oversight	Animal experiments were approved by ethics committee of Tianjin University in compliance with the law on experimental animals. The animal protocols are compliant with all relevant ethical regulations.

Note that full information on the approval of the study protocol must also be provided in the manuscript.

Flow Cytometry

Plots

Confirm that:

- [x] The axis labels state the marker and fluorochrome used (e.g. CD4-FITC).
- [x] The axis scales are clearly visible. Include numbers along axes only for bottom left plot of group (a 'group' is an analysis of identical markers).
- [ ] All plots are contour plots with outliers or pseudocolor plots.
- [ ] A numerical value for number of cells or percentage (with statistics) is provided.

Methodology

Sample preparation	MDA-MB-231 cells were seeded into 6-well plate and grown to around 80-90% confluence. Culture medium was replaced with fresh medium containing Cy5-labeled nanogels (3 µM equivalent Cy5 concentration). After incubation for indicated time (1 h, 2 h, 4 h, 6 h), the cells were washed three times with PBS and harvested by trypsin treatment. Then the harvested cells were washed twice with PBS and collected by centrifugation. Finally, the cells were re-suspended with PBS and lifted with a 300 mesh filter for flow cytometry analysis.
Instrument	BD FACSAria III flow cytometer
Software	BD FACSAria III Software
Cell population abundance	Population abundance was determined by collecting a fixed (10000) cell number for each sample.
Gating strategy	The gating strategy has been described in Supplementary information.

- [x] Tick this box to confirm that a figure exemplifying the gating strategy is provided in the Supplementary Information.
